# Probing anharmonic and heterogeneous carrier dynamics across sublattice melting in a minimal model superionic conductor

**DOI:** 10.1073/pnas.2605867123

**Published:** 2026-07-07

**Authors:** Sucharita Niyogi, Takenobu Nakamura, Genki Kobayashi, Yasunobu Ando, Takeshi Kawasaki

**Affiliations:** ^a^ Large-Scale Computational Science Research Division, D3 Center, The University of Osaka, Toyonaka 560–0043, Japan; ^b^ Department of Physics, The University of Osaka, Osaka 560–0043, Japan; ^c^ Department of Physics, Nagoya University, Nagoya 464–8601, Japan; ^d^ Department of Materials and Chemistry Materials DX Research Center, National Institute of Advanced Industrial Science and Technology, Ibaraki 305–8568, Japan; ^e^Solid State Chemistry Laboratory, Pioneering Research Institute (PRI), RIKEN, Saitama 351–0198, Japan; ^f^ Institute of Integrated Research, Institute of Science Tokyo, Kanagawa 226–8501, Japan

**Keywords:** superionic conductors, sublattice melting, solid-state batteries, dynamical heterogeneity, anharmonicity

## Abstract

Despite decades of research, the microscopic origin of sublattice melting and fast ion transport in superionic conductors remains elusive. Here, we introduce a chemically neutral minimal binary model consisting of a rigid host lattice stabilized by short-range steric repulsion and a soft carrier sublattice interacting via long-range Wigner-type forces. This contrast naturally produces distinct melting temperatures and an intermediate sublattice-melting phase in which carriers become fluidlike while the host remains crystalline. Molecular dynamics simulations identify multiple dynamical regimes–crystalline, sublattice-melt, and fully molten–marked by sharp changes in diffusivity, structural correlations, and dynamical heterogeneity. Near sublattice melting, carrier motion is strongly anharmonic and spatially heterogeneous, beyond mean-field hopping descriptions. By tuning the density, we demonstrate that sublattice melting can be continuously controlled, establishing a direct link between lattice softness, anharmonicity, and collective ion transport. Comparison with conventional long-range Coulombic models confirms that our minimal model reproduces the key dynamical signatures of superionicity, providing a unified microscopic foundation for designing mechanically robust superionic conductors.

## Significance

Why ions in some crystals flow like a liquid while the lattice remains solid is a long-standing puzzle. Using a simple, chemically neutral model of a superionic conductor, we show that the emergence of liquid-like ionic transport arises not from independent hopping but from collective, spatially heterogeneous string-like motions enabled by lattice softening through anharmonicity. By tuning particle density, we show that this cooperative mechanism can be controlled across a wide range of conditions. We further show that this behavior is robust across dimensions and systematically depends on carrier size. Because the model deliberately excludes material-specific chemistry, these results reveal a universal physical mechanism linking lattice softness, cooperative dynamics, and fast ion transport, providing general design principles for solid electrolytes.

The rapid advancement of energy storage technologies has placed rechargeable batteries at the center of modern society, powering applications from portable electronics to electric vehicles and large-scale renewable energy integration. At the core of all such battery technologies is ionic transport: ionic conductors, whether liquid or solid, are essential for efficient charge transport and directly determine battery performance, longevity, and safety. While liquid electrolytes in conventional Li-ion batteries provide high ionic mobility and performance (1), these volatile solvents are flammable, chemically reactive, and prone to leakage, making them a primary source of thermal runaway and catastrophic failure in high-energy-density devices (2). Therefore, overcoming these limitations is crucial for the safe and reliable deployment of next-generation batteries.

In this context, solid-state electrolytes, particularly superionic conductors (3–20), constitute a distinct class of materials in which ions move almost as freely as in molten salts while the host lattice retains long-range crystalline order. Their defining feature is the coexistence of two distinct sublattices: an immobile framework that maintains structural integrity and a mobile-ion sublattice that becomes disordered at elevated temperatures (21). The temperature dependence of this order–disorder transition varies widely among materials. For example, NaCl exhibits a pronounced increase in ionic conductivity only near its melting point, consistent with conventional melting behavior, whereas some superionic conductors, such as PbF_2_, show a gradual enhancement of conductivity over several hundred Kelvin, well below the melting temperature (17, 18, 22). Others, including AgI, undergo a first-order phase transition accompanied by an abrupt increase in ionic conductivity by more than three orders of magnitude. Similar melting-like transport behavior has also been reported in experiments on other charge carriers, including hydride ions (H^−^), indicating the generality of this phenomenon across some ion species (16, 20). Despite these observations, the microscopic origin of such an anomalous transition remains unresolved. Some recent analyses of layered and tunnel-type superionic conductors have shown that carrier transport is strongly influenced by many-body interactions among mobile ions, reshaping the diffusion landscape beyond simple single-particle hopping; here, Raman measurements and molecular dynamics (MD) simulations further show that short-range cation–cation repulsion modifies activation barriers, underscoring the collective nature of fast-ion motion (23). Together, these studies suggest that sublattice melting and strong anharmonic lattice fluctuations are both essential ingredients for superionic transport; however, a direct microscopic link connecting these phenomena remains to be clearly established.

To place these observations in context, sublattice melting (5, 10, 24–27) refers to a state in which the mobile ionic network becomes dynamically disordered while the host framework remains crystalline, enabling liquid-like conductivity without structural collapse. Early mean-field models (25) attributed this transition to a balance between defect formation energy and configurational entropy, predicting disorder within one ionic sublattice prior to complete melting. However, their defect-based mean-field framework precludes access to real-space ion dynamics, spatial correlations, and anharmonic lattice effects central to superionic transport. Experimental studies on CuI provided early dynamical evidence for sublattice melting: NMR measurements showed anomalously enhanced relaxation near the superionic transition, consistent with a molten copper sublattice within an ordered iodide framework (10, 26). Subsequent theoretical treatments (24) described the precursor defect proliferation using a cube-root dependence of the defect chemical potential, reproducing the premelting characteristics observed in AgI and PbF_2_. More recently, analogous phenomena have been realized in asymmetric colloidal crystals (5), where smaller charged particles delocalize within a crystalline matrix of larger oppositely charged spheres, and in ultrasmall copper selenide clusters exhibiting liquid-like cationic sublattices even at ambient temperature (28). Complementary experimental studies have since directly visualized this selective sublattice melting (29), reaffirming its universality in ion-conducting solids. Despite these advances, the microscopic origin of such selective disorder remains poorly understood, as most existing descriptions rely on mean-field or defect-based frameworks that neglect correlated particle motion and its feedback on the lattice vibrational landscape. An instructive analogy is provided by colloidal Wigner crystals, where crystalline order is maintained despite large-amplitude particle fluctuations due to significant configurational entropy (30), highlighting the role of anharmonic dynamics within an ordered framework.

Selective sublattice disorder reshapes the vibrational potential energy landscape, introducing anharmonicity that enables nonlinear energy exchange and mode coupling between phonons (31–33). Such anharmonicity effectively softens the lattice and lowers local activation barriers for ionic motion. Early studies on superionic AgI (31) proposed that Ag^+^ ions undergo strongly anharmonic, semi-liquid-like thermal vibrations that extend toward neighboring interstitial sites. This anharmonic motion can overcome local potential barriers and promotes ionic hopping, while appearing in diffraction as a pseudostatic occupation of interstitial sites rather than true site disorder. High-pressure analyses (32) further showed that harmonic or mean-field descriptions fail to capture melting behavior driven by such vibrations. More recent first-principles and spectroscopic studies on sodium-ion conductors (33) demonstrated that strong host-ion anharmonic coupling induces order–disorder transitions with soft modes persisting across the phase change, directly linking lattice dynamics to fast ionic conduction. Nevertheless, establishing a unified microscopic description that connects anharmonic lattice dynamics with emergent collective transport behavior remains an important challenge.

Together, these studies suggest that sublattice melting and anharmonicity are not independent phenomena but mutually reinforcing aspects of the same underlying physics, in which selective ionic disorder feeds back into lattice softening, and vice versa. In particular, recent work on Li_10_GeP_2_S_12_ has shown that Li^+^ conduction proceeds via correlated and cooperative migration of densely packed ions (9), explicitly invoking the concept of dynamical heterogeneity through the coexistence of fluid-like, highly mobile regions and immobile rigid domains. This coexistence of mobile and immobile regions is a well-known hallmark of glass-forming systems and other dynamically heterogeneous materials. More broadly, dynamical heterogeneity is a central concept in glass-forming systems, where it underlies transport anomalies and cooperative motion (34–41). At the microscopic level, such heterogeneous dynamics have been linked to localized anharmonic vibrational fluctuations and soft quasilocalized nonphononic modes that promote collective rearrangements (42, 43). In this light, dynamical heterogeneity offers a natural conceptual bridge between sublattice melting and anharmonicity in superionic conductors: spatial variations in local anharmonicity amplify mobility contrasts, giving rise to cooperative, string-like dynamics beyond mean-field descriptions, as observed in glass-forming liquids where enhanced vibrational fluctuations precede collective string-like rearrangements (44). The extent to which analogous vibrational-heterogeneity couplings govern fast-ion transport in crystalline solids remains an open question.

To elucidate this connection, we employ a minimal and tractable model to systematically probe how temperature and interactions control sublattice melting and collective carrier motion. This approach allows us to identify the conditions under which liquid-like regions emerge within an otherwise rigid lattice, quantify correlated ion dynamics beyond mean-field descriptions, and explore the emergence of superionic conduction. By linking lattice softness, anharmonicity, and dynamical heterogeneity, this framework provides a simplified route to understand fast-ion transport and design mechanically robust, high-performance superionic conductors.

## Results

Our analysis focuses on three key mechanisms–selective sublattice melting, structural heterogeneity, and anharmonicity–to elucidate ion-transport dynamics in two-dimensional (2D) systems. In 2D, true long-range translational order is prohibited at finite temperature by the Hohenberg–Mermin–Wagner theorem (45–50), although the orientational order may persist. Our conclusions do not rely on strict translational long-range order; rather, they concern the emergence of liquid-like transport within an ordered host through enhanced anharmonicity and spatially heterogeneous, string-like motion. 2D systems are therefore used primarily for clarity and visualization, while corresponding three-dimensional (3D) results are presented in *SI Appendix*.

### Microscopic Configurations and Sublattice Disorder.

To investigate the microscopic dynamics of the 2D host–carrier system, we employ our nonadditive potential (NAP) model systems in which the host and carrier form stable crystals at low temperatures. The choice of such a system reflects the long-range nature of the interaction potential, analogous to the Coulomb force (see *Materials and Methods* for details). Under these conditions, low-density carriers naturally organize into ordered patterns at low temperatures, forming a well-defined crystalline state. As the temperature increases, the host lattice remains crystalline while the carrier sublattice progressively melts. This configuration provides a mechanically stable reference state from which the subsequent thermal evolution and melting processes can be systematically explored, ensuring that the high-temperature dynamics originate from physically meaningful particle arrangements established at low temperatures.

**Fig. 1.**
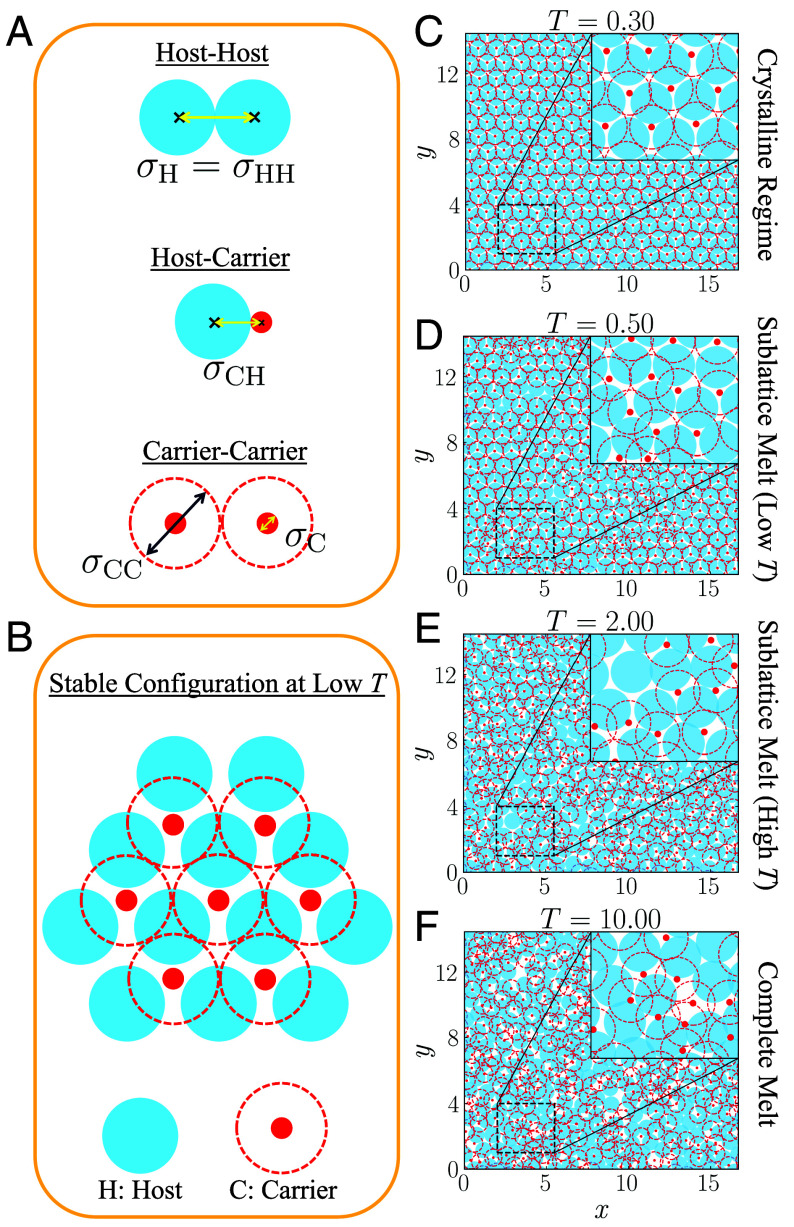
Structural representations of the host–carrier in 2D NAP model. (*A*) Schematic representation of the interaction scheme between interparticle pairs: The host–host interaction length is denoted as $\sigma_{H}=\sigma_{\mathrm{HH}}$, the host–carrier interaction length as $\sigma_{\mathrm{CH}}$, the nonadditive interaction length $\sigma_{\mathrm{CC}}$ between carrier–carrier is represented by the red dotted line, and the effective–carrier interaction length is chosen to be $\sigma_{C}$ (see *Materials and Methods* for more details). (*B*) Low-temperature configuration obtained using a Wigner-type interaction, illustrating the ordered host lattice (blue) with carrier particles (red) occupying energetically favorable interstitial sites. (*C*–*F*) Representative snapshot configurations illustrating crystalline, sublattice-melt, and fully molten states across increasing temperature (*Top* to *Bottom*) for area packing fraction ϕ=0.85. For clarity, each snapshot includes an *Inset* highlighting a magnified region of the configuration, emphasizing local structural arrangements and carrier environments.

Fig. 1 illustrates the structural framework underpinning our 2D NAP model. The host and carrier particles interact through nonadditive pair potentials that define distinct effective length scales, as summarized in Fig. 1*A*. These interaction parameters are coarse-grained, effective quantities that encode collective lattice stiffness and carrier confinement, rather than specific microscopic chemistry. At low temperature (*T*) (Fig. 1*B*), the interactions stabilize a crystalline arrangement in which carriers occupy well-defined interstitial positions within the host lattice–a reference configuration from which all subsequent temperature-dependent transformations are analyzed. To provide a broader picture, Fig. 1*C*–*F* present representative structural snapshots at different temperatures, where one can clearly observe the progressive loss of order. In the most stable, low-temperature state (T=0.30 in Fig. 1*C*; for units see *Materials and Methods*), both host and carrier particles exhibit a well-preserved crystalline symmetry. Upon heating, a sublattice-melting transition emerges (Fig. 1 *D* and *E*). At the lower bound of this regime (T=0.50), partial disordering of the carrier sublattice is observed–regions of crystalline order coexist with melted domains–while the host lattice remains intact. At a higher temperature (T=2.00), the carrier sublattice becomes fully disordered, yet the host lattice preserves its crystalline arrangement. This indicates that both states lie within the sublattice-melting regime where only the carrier network loses structural order. At sufficiently high temperatures (T=10.00 in Fig. 1*F*), both host and carrier particles lose positional order, corresponding to complete melting of the system.

**Fig. 2.**
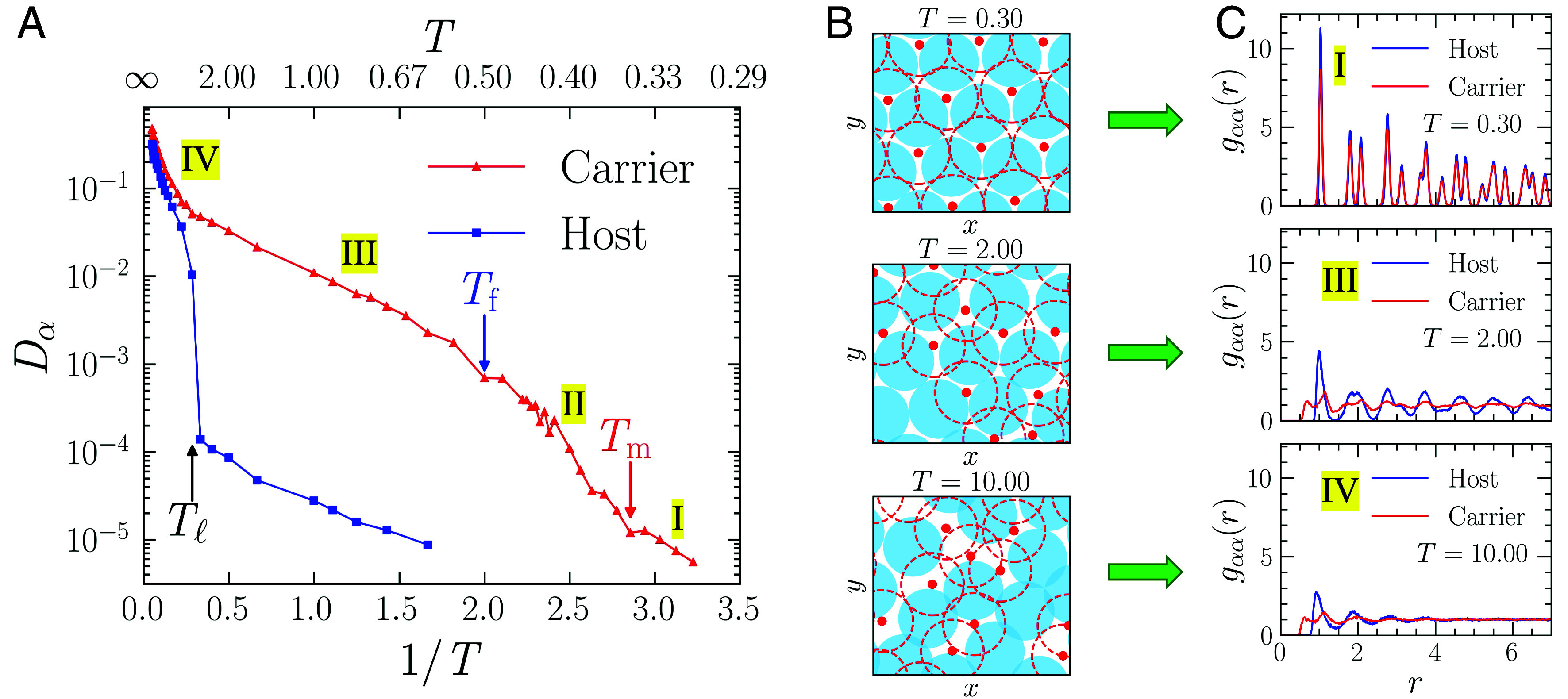
Selective sublattice melting and structural evolution in 2D NAP model for ϕ=0.85. (*A*) Schematic representation of the diffusivity $D_{\alpha }$ (α∈{C,H}, where C: Carrier and H: Host) as a function of inverse temperature 1/T, illustrating four distinct regimes: (I) crystalline, (II) onset of sublattice melting, (III) sublattice melting, and (IV) full melting. Three characteristic temperatures $T_{m}(∼$0.35), $T_{f}(∼$0.50), and $T_{\ell }(∼$3.50) are indicated, corresponding to the onset of sublattice melting, the freezing of carrier motion, and the liquid-like regime, respectively. (*B*) Representative snapshots illustrating structural evolution across these regimes. (*C*) Radial distribution functions $g_{\alpha \alpha }\left(r\right)$ corresponding to each regime. Region III shows disordered carrier sublattice coexisting with ordered host lattice, evidencing selective carrier sublattice melting.

To quantify particle mobility and capture the key characteristics underlying this phenomenon, we computed the mean-squared displacement (MSD) over time of the carrier and host particles separately (*Materials and Methods* and Fig. 4*A*). The long-time diffusive behavior of the MSD was then used to calculate the self-diffusion coefficient $D_{\alpha }$ (α∈{C,H}, where C: Carrier and H: Host) using the Einstein relation (*Materials and Methods*). This analysis was performed separately for both carrier and host particles, allowing us to directly compare their respective mobilities and the influence of temperature on their transport dynamics. In Fig. 2*A*, we identify four distinct dynamical regimes characterized by changes in the temperature dependence of the diffusivity. Importantly, these regimes are not introduced as empirical classifications, but emerge from a progressive change in the underlying microscopic dynamics. At low temperatures (regime I), both host and carrier particles exhibit nearly harmonic vibrations around their equilibrium positions. In this regime, particle motion is dominated by independent, thermally activated hopping over well-defined local energy barriers, leading to Arrhenius behavior with comparable slopes for both species. As the temperature increases toward the onset of sublattice melting (regime II), anharmonic lattice fluctuations become significant. These fluctuations locally distort the confining potential experienced by the carriers, effectively softening the energy landscape and reducing the activation barriers for migration. As a result, particle motion begins to deviate from independent hopping and increasingly involves correlated displacements. In the sublattice-melting regime (regime III), these effects become dominant. The enhanced anharmonicity leads to the formation of spatially heterogeneous regions with reduced local stiffness, which act as pathways for cooperative carrier motion. Transport is therefore no longer governed by a single-particle activation process, but by collective, string-like rearrangements involving multiple carriers. This crossover is reflected in the marked enhancement of carrier diffusivity and the breakdown of a simple Arrhenius description. At even higher temperatures (regime IV), once the host lattice loses structural integrity, the system transitions to a fully molten state. In this regime, both species exhibit homogeneous, liquid-like dynamics, and transport is governed by standard kinetic mechanisms rather than by cooperative processes within a solid framework. Taken together, these results demonstrate that the temperature-dependent transport behavior arises from a continuous evolution of the energy landscape: Increasing anharmonicity reduces effective activation barriers and promotes cooperative dynamics, ultimately leading to sublattice melting and liquid-like transport. These findings are in good agreement with previous experimental observations that reported similar melting signatures in related systems (16, 20, 51). A direct comparison between our 2D NAP model predictions and available experimental trends is provided in *SI Appendix*.

To aid the interpretation of the transport regimes, Fig. 2*B* shows magnified cross-sectional views at representative temperatures (T=0.30, 2.00, and 10.00), corresponding to the crystalline, sublattice-melted, and fully molten states already discussed in Fig. 1*C*, *E*, and *F*. These views highlight the progressive loss of order in the carrier sublattice while the host lattice remains intact in the sublattice-melting regime, followed by complete disordering of both components at high temperature.

To quantitatively characterize these structural correlations, we calculate the radial distribution function (RDF), $g_{\alpha \alpha }\left(r\right)$, for both carrier and host particles (*Materials and Methods*). As shown in Fig. 2*C*, the RDF exhibits sharp periodic peaks at low temperature (region I), reflecting the long-range crystalline order of both the host and the carrier particles. In the intermediate regime (region III), the contrast becomes evident: The RDF of the host particles retains pronounced peaks even at higher temperatures (e.g., T=2.00), while that of the carriers broadens into liquid-like correlations, corroborating the sublattice melting identified from the snapshots. At very high temperatures (e.g., T=10.00 in region IV), both sublattices lose positional order, and the RDFs of hosts and carriers display the characteristic liquid-like structure with damped oscillations. A detailed analysis of the temperature evolution of the carrier and host RDFs, including the emergence of excess short-range features associated with activated carrier hopping, is provided in *SI Appendix*.

### Dynamical Aspect of Sublattice Melt.

**Fig. 3.**
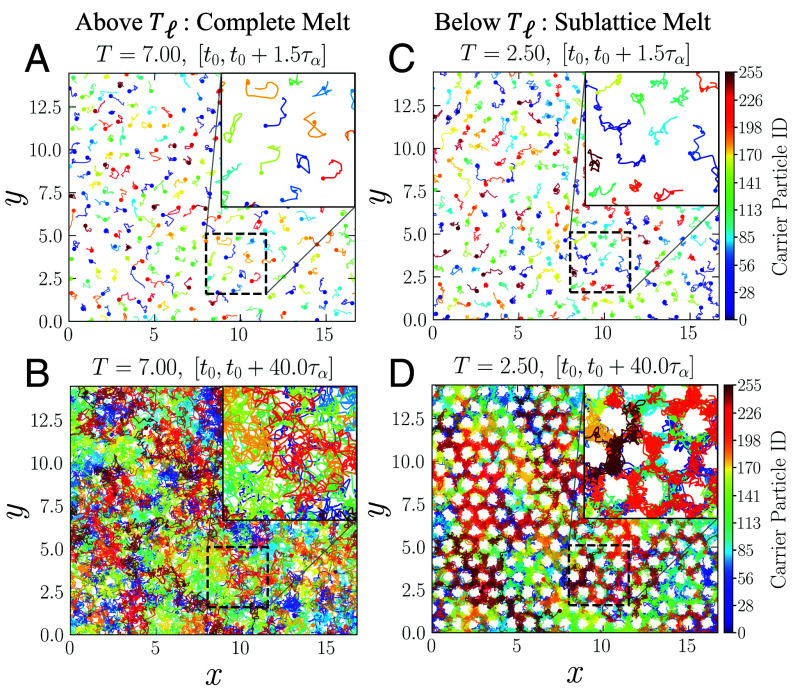
Trajectory of carriers at different time intervals and temperature regimes for our 2D NAP model for ϕ=0.85. Panels (*A*–*D*) show carrier trajectories within region IV-III of the diffusivity plot (as shown in Fig. 2*A*), corresponding to the complete melt and sublattice melting regime. Panels (*A* and *B*) present trajectories above the melting temperature of host within region IV, beyond the sublattice-melting regime, while panels (*C* and *D*) correspond to the higher-temperature side of the sublattice-melting regime (region III). Panels (*A* and *C*) correspond to shorter observation time windows, whereas panels (*B* and *D*) show the dynamics over longer time windows. Carrier particle indices (ID) are color-coded as indicated by the colorbar on the right-hand side. For corresponding movies of these trajectories, see *SI Appendix*.

While the previous analyses characterize the structural and transport properties of the system through static configurations and diffusivity measurements, a direct visualization of particle trajectories provides additional insight into the dynamical aspects of sublattice melting. To this end, we trace the trajectories of representative carrier particles within the host lattice and map their motion across distinct time windows and temperature regimes. Fig. 3 presents the carrier trajectories at representative temperatures for area packing fraction ϕ=0.85, illustrating the evolution of carrier dynamics across the sublattice-melting transition. At high temperatures, e.g. T=7.00 (Fig. 3 *A* and *B*), both the host lattice and the carrier sublattice are completely melted. Consequently, the trajectories exhibit homogeneous liquid-like motion at both short ($t=1.5\tau_{\alpha }$) and long ($t=40.0\tau_{\alpha }$) time windows, where $\tau_{\alpha }$ denotes the structural relaxation time corresponding to each temperature. Upon lowering the temperature from the fully molten state into the sublattice-melting regime (Fig. 3 *C* and *D*), the carrier trajectories begin to reflect the underlying crystalline environment imposed by the host lattice. While the short-time motion remains relatively localized, the long-time trajectories form a characteristic honeycomb-like pattern associated with diffusion through the interstitial pathways of the hexagonal host structure. This behavior indicates that the host lattice remains dynamically stable while simultaneously enabling long-range carrier transport through interconnected diffusion channels. This apparent honeycomb pattern reflects carrier motion along the interstitial network defined by the host lattice, rather than hopping through host sites (see *SI Appendix* for a detailed visualization), demonstrating how the partially ordered host framework constrains and guides carrier diffusion.

### Dynamical Heterogeneity in Solid Ionics.

**Fig. 4.**
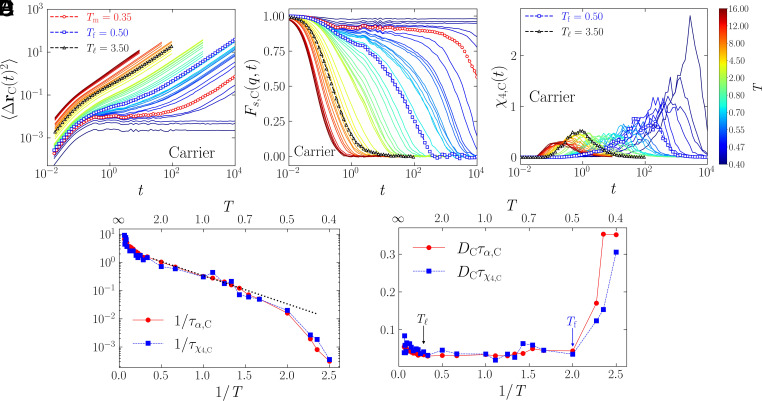
Microscopic dynamics and the extent of dynamical heterogeneity in our 2D NAP model for ϕ=0.85. (*A*–*C*) Carrier dynamics are characterized by mean-squared displacement (MSD), self-intermediate scattering function $F_{s,C}\left(q,t\right)$, and four-point susceptibility $\chi_{4,C}\left(t\right)$ at different temperatures (indicated by color bar on the right). The characteristic temperatures $T_{m}(∼$0.35), $T_{f}(∼$0.50), and $T_{\ell }(∼$3.50)–marking the onset of sublattice melting, the freezing of carrier motion, and the liquid-like regime, respectively–are indicated by red circles, blue squares, and black triangles connected with dotted lines. (*D* and *E*) Inverse-temperature dependence of the Stokes–Einstein (SE) ratios $D_{C}\tau_{\alpha ,C}$ and $D_{C}\tau_{\chi_{4,C}}$, highlighting pronounced SE violation at low temperatures (*E*). A guideline in (*D*) emphasizes the slope change near sublattice-melting transition.

To elucidate the macroscopic dynamics in the system, we analyzed several dynamic observables across different temperature regimes, as summarized in Fig. 4. The panels *A*–*C* show the MSD of the carrier particles, the self-intermediate scattering function $F_{s,C}\left(q,t\right)$, and the four-point susceptibility $\chi_{4,C}\left(t\right)$, respectively (*Materials and Methods*). For reference, three characteristic temperatures–$T_{m}(∼$0.35), $T_{f}(∼$0.50), and $T_{\ell }(∼$3.50)–identified from the diffusivity behavior in Fig. 2, are marked in all panels. These represent, respectively, the onset of sublattice melting, the freezing of carrier motion, and the liquid-like regime.

The MSD data (Fig. 4*A*) reveal that at low temperatures near $T_{m}$, carriers exhibit pronounced caged motion, which gradually weakens with increasing temperature and eventually dissolves above $T_{f}$, leading to fully diffusive dynamics. Correspondingly, the self-intermediate scattering function $F_{s,C}\left(q,t\right)$ (Fig. 4*B*), which measures the temporal correlation of particle positions at wavevector q, exhibits the emergence of a plateau around $T_{f}$, signaling slow structural relaxation associated with carrier localization. At higher temperatures, $F_{s,C}\left(q,t\right)$ decays rapidly without any plateau, consistent with liquid-like mobility.

The four-point susceptibility for carriers $\chi_{4,C}\left(t\right)$ (Fig. 4*C*), which quantifies fluctuations in particle mobility and thus the extent of cooperative motion, captures the growth and suppression of correlated dynamics (37, 38, 52). At low temperatures, $\chi_{4,C}\left(t\right)$ exhibits large and sharply peaked maxima at long times, indicating strong dynamical heterogeneity and persistent cooperative motion within the ordered carrier sublattice. As the temperature increases toward the sublattice-melting regime near $T_{f}$, the peak amplitude decreases rapidly and shifts to shorter times, reflecting the progressive loss of long-lived spatial correlations as the sublattice destabilizes. At still higher temperatures, only weak and broad peaks remain, consistent with increasingly homogeneous relaxation dynamics. Thus, the strong suppression of $\chi_{4,C}\left(t\right)$ across the melting regime provides a microscopic signature of the crossover from heterogeneous cooperative transport to homogeneous diffusive motion.

To probe the connection between microscopic dynamics and transport, we examined the inverse relaxation times, $1/\tau_{\alpha ,C}$ and $1/\tau_{\chi_{4,C}}$, as a function of inverse temperature 1/T (Fig. 4*D*) (see *Materials and Methods* for the definition of these time scales). Both quantities exhibit nearly Arrhenius behavior at high temperatures, but deviate sharply near $T_{f}$, indicating the onset of heterogeneous dynamics consistent with the MSD, $F_{s,C}\left(q,t\right)$, and $\chi_{4,C}\left(t\right)$ results. In the Arrhenius regime, relaxation is governed by an approximately fixed activation barrier, such that the characteristic relaxation time or diffusivity follows a single Arrhenius slope. This implies that particles cross comparable energy barriers irrespective of the instantaneous configuration of their neighbors, a hallmark of weakly correlated dynamics, typical of normal liquids or dilute systems. However, near the sublattice-melting transition, particle motion becomes increasingly cooperative and dynamically heterogeneous, with mobile and immobile regions coexisting in space and time. Structural relaxation therefore involves correlated rearrangements rather than isolated hopping events, leading to deviations from simple Arrhenius behavior in the relaxation dynamics. By contrast, purely local, single-particle hopping dynamics (41) would preserve a nearly constant activation barrier and thus Arrhenius behavior even near the transition. Accordingly, the emergence of correlated motion naturally implies a breakdown of the Stokes–Einstein (SE) relation.

**Fig. 5.**
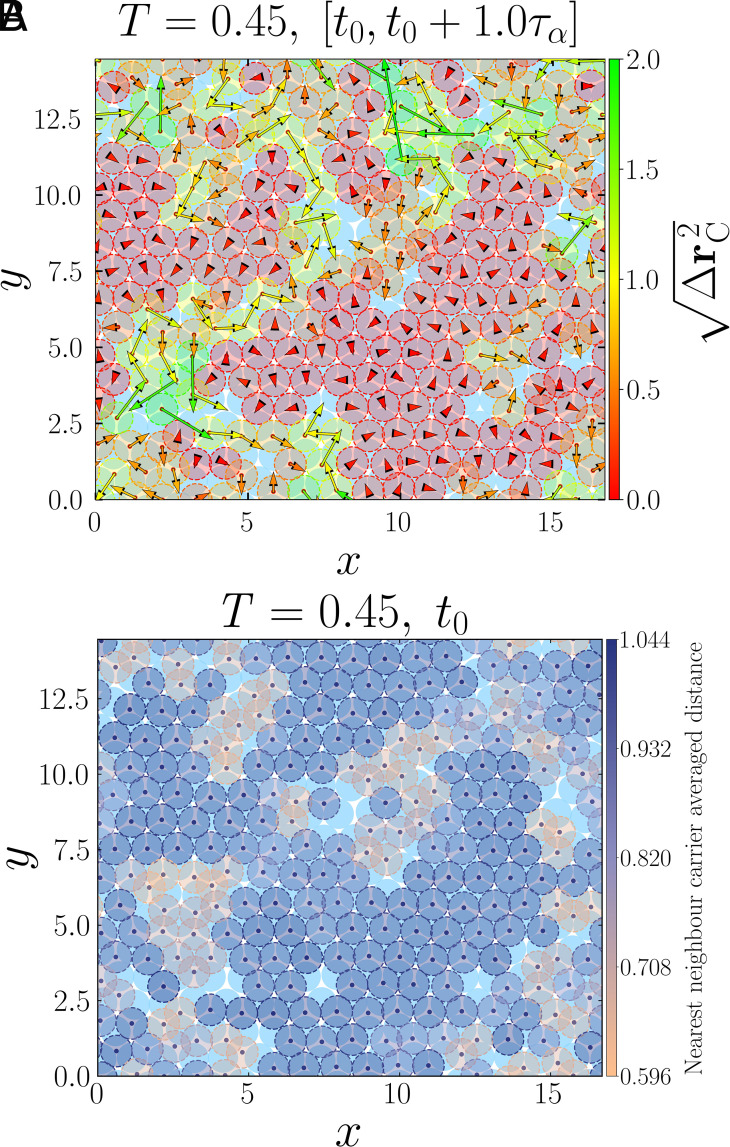
Interplay between dynamic and static heterogeneity near the sublattice-melting point in our 2D NAP model for ϕ=0.85. (*A*) Spatial map of root mean-squared displacement of carriers over $t\in [t_{0},\;t_{0}+\tau_{\alpha }]$ at T=0.45, showing coexisting liquid-like and immobile crystalline domains. Displacement vectors in (*A*) further reveal string-like cooperative motion and hopping pathways within liquid-like regions, contrasted with localized, heterogeneous vibrations in crystallized domains. (*B*) Static configuration at $t_{0}$, colored by nearest carrier–carrier averaged distance, revealing anharmonic regions that align with the dynamically mobile zones in (*A*). Together, the two panels show that dynamical heterogeneity emerges from underlying static distortions of the carrier sublattice.

The corresponding SE ratios $D_{C}\tau_{\alpha ,C}$ and $D_{C}\tau_{\chi_{4,C}}$ (Fig. 4*E*) remain nearly constant at high T, show a modest reduction near $T_{\ell }$, and increase sharply below $T_{f}$. This violation of the Stokes–Einstein relation is a hallmark of growing dynamical heterogeneity: Structural relaxation slows markedly as correlated regions develop, whereas diffusion remains comparatively fast due to cooperative carrier motion. This violation of SE relation is consistent with nontrivial carrier–carrier correlations, which are commonly reflected in deviations of the Haven ratio from unity in heterogeneous transport regimes (53). The resulting decoupling between the diffusion and relaxation time scales becomes pronounced once heterogeneous dynamics are long-lived (54), signaling the onset of sublattice melting. While these signatures establish the presence of dynamical heterogeneity (39, 41) from a macroscopic transport perspective, they do not resolve its microscopic origin. To directly probe the spatiotemporal structure of heterogeneous carrier motion near $T_{f}$, we therefore examine particle-resolved displacements and local mobility patterns in Fig. 5.

To complement the dynamical observables discussed above, Fig. 5 *A* and *B* presents direct evidence of how structural and dynamical heterogeneity emerge near the sublattice-melting transition. Fig. 5*A* maps the spatial distribution of carrier particle mobility through $\sqrt{\Delta r_{C}^{2}}$ over the interval $\left[t_{0},t_{0}+\tau_{\alpha }\right]$ at T=0.45, further enhanced by displacement-vector arrows whose length and color encode the magnitude of particle motion. These arrows reveal not only the coexistence of liquid-like and immobile domains, but also the presence of concerted hopping events, manifested as string-like cooperative motion and correlated intermittent jumps within the mobile regions. Such collective displacements indicate that ion transport proceeds via cooperative rearrangements rather than independent single-particle hopping. This coexistence of mobile pathways and localized particles reflects pronounced dynamical heterogeneity at low temperature, which progressively diminishes with increasing temperature (*SI Appendix*). Notably, enhanced mobility is also observed near the edges of crystallized domains, reflecting pronounced local fluctuations of the carrier environment. In contrast, the crystallized (red colored) regions predominantly display short, irregular, and spatially heterogeneous displacement vectors, indicative of confined yet nonuniform vibrational dynamics. The corresponding static snapshot in Fig. 5*B*, colored by the local carrier–carrier nearest-neighbor averaged distance, exhibits a strikingly similar spatial pattern: Particles in locally compressed, liquid-like environments (peach colored) are spatially collocated with the dynamically mobile regions identified in Fig. 5*A*, whereas particles in more expanded, ordered environments (navy blue colored) coincide with dynamically frozen domains. Such a correspondence between structural softness and enhanced mobility closely parallels observations in glass-forming systems, where regions of local densification serve as loci of dynamical heterogeneity (39, 55). The emergence of such spatial heterogeneity is consistent with the breakdown of carrier hyperuniformity upon approaching $T_{m}$, reflecting enhanced long-wavelength density fluctuations (*SI Appendix*) (56). Notably, the peach-colored regions correspond to carrier particles transiently occupying interstitial positions within the host lattice. As previously proposed for superionic AgI, transient occupation of interstitial sites leads to strongly anharmonic vibrational motion rather than simple harmonic oscillations about lattice sites (31). More broadly, recent studies in amorphous solids have shown that such emergent anharmonicity—manifested through low-frequency vibrational anomalies and instantaneous unstable modes—signals the proliferation of shallow, low-barrier sectors in the potential-energy landscape (57–59). These insights may provide a natural microscopic framework for interpreting fast ionic transport in superionic conductors and its connection to the heterogeneous carrier dynamics observed in Fig. 5. This directly motivates a quantitative analysis of lattice anharmonicity as the system approaches the sublattice-melting regime, which we pursue in the next section.

### Growth of Anharmonicity Near the Sublattice Melting Point.

To quantify the anharmonic vibrational state revealed above, we examine direct measures of lattice stability. In particular, we focus on the Debye–Waller factor, $u_{\alpha }^{2}$, defined as the mean-squared displacement of particles within their local cages, providing a measure of the vibrational amplitude that reflects local stiffness and the degree of anharmonicity in the lattice. In practice, $u_{\alpha }^{2}$ is extracted from the plateau value of the Lindemann index. A detailed analysis based on the Lindemann index is presented in *SI Appendix*.

**Fig. 6.**
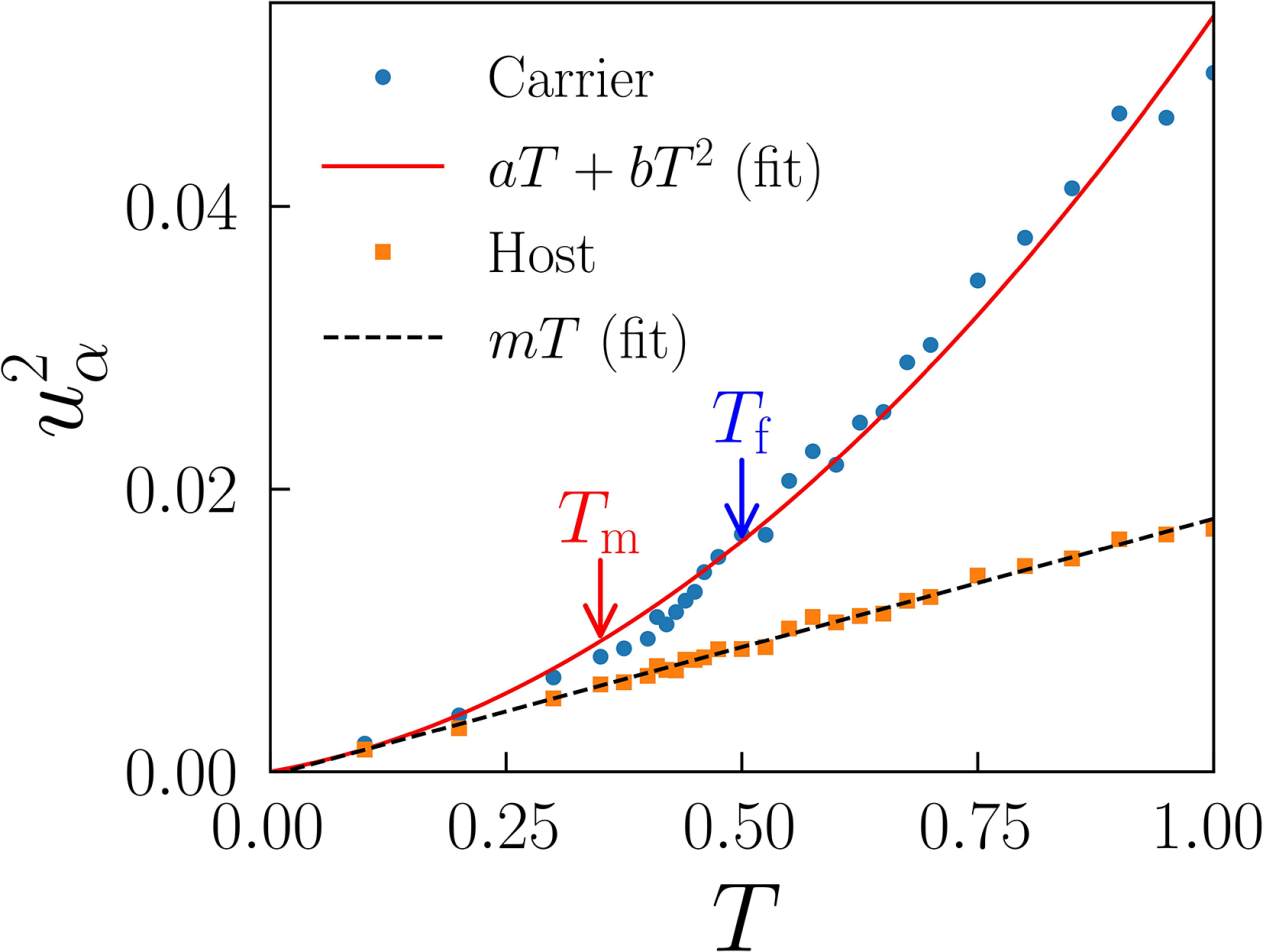
Anharmonic vibrational behavior from the Debye–Waller factor in our 2D NAP model for ϕ=0.85. Distinct anharmonic behavior is evident in the Debye–Waller factor: The host exhibits a linear dependence (black dotted line), indicating harmonic cage vibrations, whereas the carrier shows clear anharmonic effects (red solid line) with two distinct slopes, reflecting the increasing significance of nonlinear contributions near the onset of sublattice melting.

For the host particles (see Fig. 6), $u_{H}^{2}$ increases linearly with temperature, $u_{H}^{2}\propto T$, indicating that their dynamics are governed by harmonic cage vibrations, as expected for an elastically stable lattice (60). We further find that the host lattice exhibits a density-dependent deviation from harmonic behavior: It remains approximately harmonic at high densities, whereas at lower densities $u_{H}^{2}$ develops a clear nonlinear temperature dependence, indicating lattice softening and the onset of anharmonicity (see *SI Appendix* for more details).

In contrast, the carrier particles exhibit a pronounced nonlinear temperature dependence of the form $u_{C}^{2}=aT+bT^{2}$ (60), with the quadratic contribution becoming increasingly significant as the system approaches the sublattice melting point. Within standard statistical-mechanical descriptions, such nonlinear behavior arises from anharmonic terms in the effective potential, signaling local softening and strongly anharmonic carrier fluctuations even while the host lattice remains elastically stable.

These results demonstrate that anharmonicity grows continuously upon approaching sublattice melting: The host retains predominantly harmonic, lattice-governed vibrations at high densities, whereas the carrier particles display pronounced nonlinear responses, reflecting the breakdown of harmonic confinement and the emergence of dynamically heterogeneous motion.

### Controlling the Sublattice Melting.

**Fig. 7.**
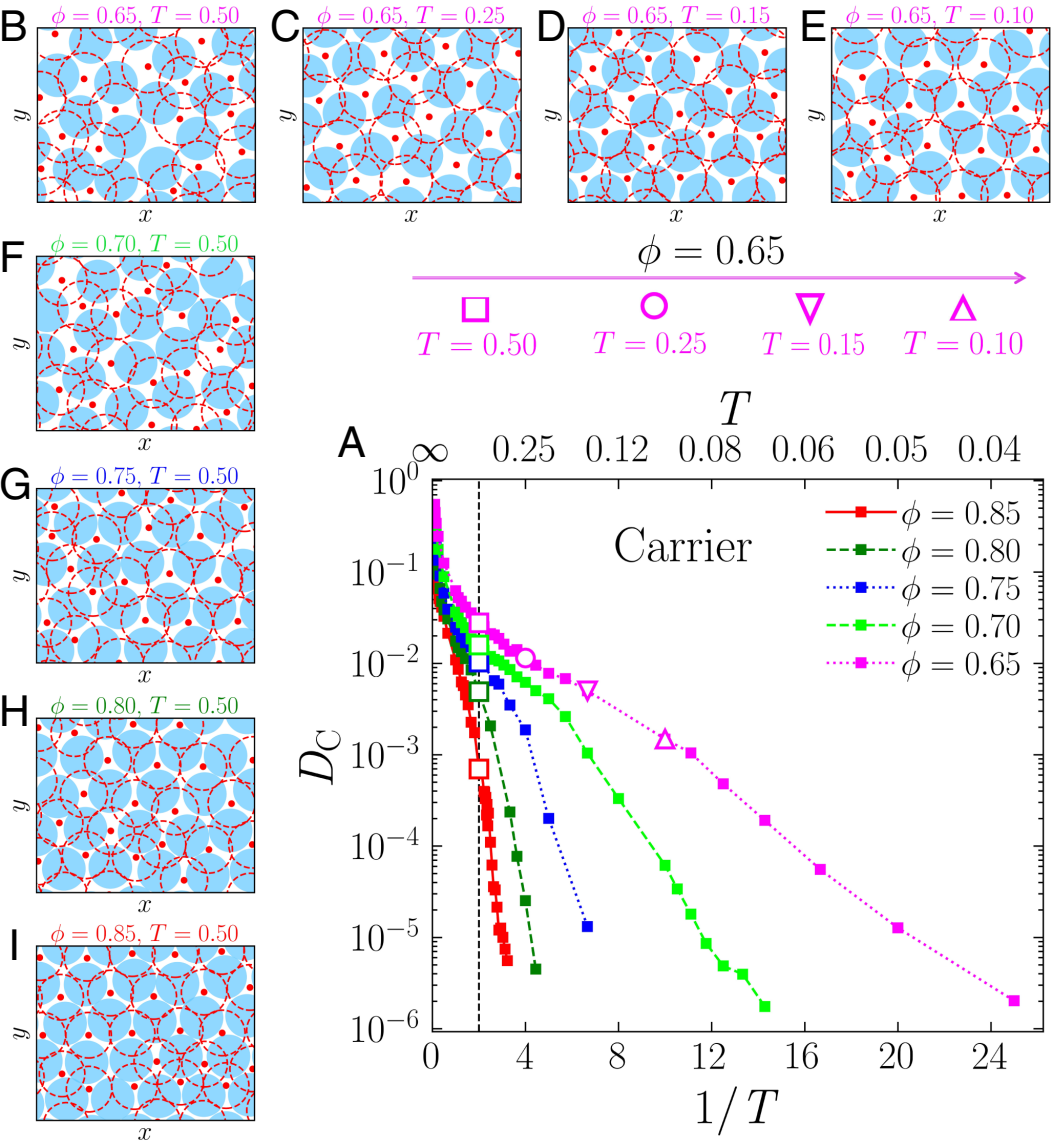
Density dependence of the diffusivity in our 2D NAP model. (*A*) Temperature dependence of diffusivity of carriers ($D_{C}$) as a function of inverse temperature (1/T) for different area packing fractions including ϕ=0.85 (used for all previous results). The results demonstrate that tuning the packing fraction effectively controls the onset of sublattice melting. With decreasing packing fraction, the transition becomes smoother, and distinct slope changes are observed across the five packing cases, indicating different dynamical regimes. Panels (*B*–*E*) correspond to ϕ=0.65 at decreasing temperatures (T=0.50,0.25,0.15,0.10), showing progressive localization of carriers upon cooling. Panels (*B* and *F*–*I*) show configurations at T=0.50 [follow the black dashed line in (*A*)] for increasing densities (ϕ=0.65−0.85), demonstrating how higher packing suppresses anharmonic carrier motion and stabilizes the ordered sublattice. Colors are matched across diffusivity curves and corresponding configurations for each packing fraction.

We next examine how the onset of sublattice melting varies with density, characterized by the packing fraction in 2D (Fig. 7). The temperature dependence of the carrier diffusivity, $D_{C}$, shows that decreasing packing fraction broadens the crossover between solid-like and fluid-like behavior.

This behavior indicates that the structural coherence of the host lattice weakens as density is reduced, consistent with a density-dependent lattice response in which lower densities exhibit earlier deviations from harmonic behavior, reflecting enhanced lattice softness that promotes more spatially extended and weakly correlated carrier dynamics. In contrast, higher densities maintain a more rigid lattice, leading to localized, heterogeneous motion and suppressed long-wavelength density fluctuations (*SI Appendix*).

Consistent with this trend, both the host and carrier dynamics become increasingly anharmonic at lower densities (Fig. 7*A*–*I*), reflecting the progressive softening of the lattice. This softening shifts the onset of sublattice melting to lower temperatures, facilitates carrier diffusion, and reduces the degree of dynamical heterogeneity (*SI Appendix*).

### Generality of Dynamical Features: α-AgI.

**Fig. 8.**
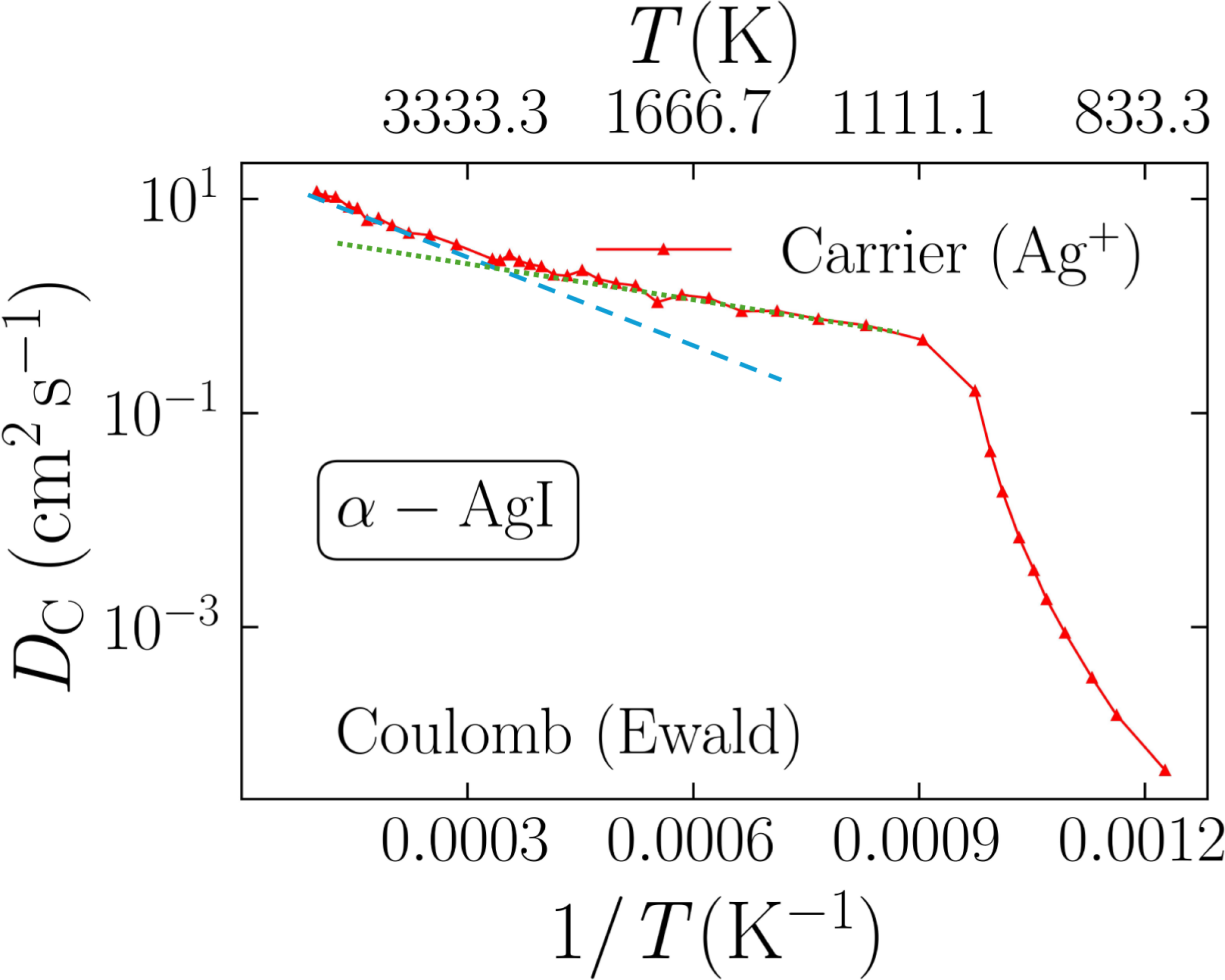
Diffusivity of 3D α-AgI (FUH) model as a function of inverse temperature. Results from molecular dynamics simulations with Coulomb interactions treated via Ewald summation. Changes in slope indicate distinct dynamical regimes within the superionic phase, consistent with sublattice melting and the onset of diffusive motion, analogous to the behavior shown in Figs. 2*A* and 7*A*.

To assess the generality of the dynamical features observed in our minimal 2D NAP model, we performed molecular dynamics simulations of 3D α-AgI using a more realistic interaction potential (FUH model) that combines short-range repulsion with long-range Coulomb interactions treated via the Ewald summation method (61) (*Materials and Methods*). The resulting temperature dependence of the diffusivity (Fig. 8) exhibits qualitative features closely resembling those of our 2D NAP model (Figs. 2*A* and 7*A*). Importantly, although the experimental AgI system exhibits a first-order structural transition, the dynamical behavior within the superionic phase remains consistent with that identified in our minimal model. In particular, distinct transport regimes associated with sublattice melting and fully developed diffusive motion, together with the crossover in diffusivity reflected by changes in slope in the Arrhenius representation, are observed independently of whether the underlying transition is continuous or discontinuous. This indicates that the microscopic transport mechanism–namely, the emergence of cooperative carrier motion driven by anharmonic lattice fluctuations–is not tied to the nature of the phase transition itself. Rather, the transition sharpness reflects thermodynamic aspects of lattice reconstruction, whereas the transport behavior is governed by dynamical processes within the superionic phase.

Because the 2D NAP model does not incorporate lattice reconstruction, it is not intended to reproduce the discontinuous structural transition itself, but rather the universal dynamical features of superionic transport.

## Summary

We have investigated a minimal 2D binary mixture model of a superionic conductor to disentangle three intertwined ingredients of fast ion transport–sublattice melting, dynamical heterogeneity, and anharmonicity–and to clarify how the density governs their emergence. The 2D NAP model captures selective sublattice melting within a chemically neutral, coarse-grained framework. Host (H) particles interact via short-range steric repulsion, forming a rigid lattice, whereas carrier (C) particles interact through much softer, long-range Wigner-type forces that promote collective delocalization. This asymmetry in interaction range naturally produces distinct melting temperatures for the two sublattices, enabling a selective carrier-melting phase where the host remains ordered while the carrier sublattice becomes fluid-like (Fig. 1). Brownian dynamics was used for equilibration, followed by long microcanonical molecular dynamics simulations to capture intrinsic dynamics (*Materials and Methods*).

Snapshots and radial distribution functions reveal clear selective disordering: The carrier sublattice loses positional order while the host remains crystalline (Figs. 1 and 2). Diffusivity-temperature plots identify four regimes (Fig. 2*A*): (I) crystalline, (II) onset of sublattice melting, (III) a broad sublattice-melting region with mobile carriers in an ordered host, and (IV) full melting. Diffusivity of carriers evolves smoothly across regime II, whereas the host shows a sharp discontinuity, consistent with a first-order-like transition (Fig. 2*A*). Carrier trajectories (Fig. 3) visualize these regimes. At high T, motion is homogeneous and isotropic. Within regime II, carriers move cooperatively along host-lattice-aligned pathways forming transient honeycomb-like networks, while at lower T they exhibit heterogeneous motion, alternating between localized and collective migration. These patterns confirm that sublattice melting arises from correlated, anharmonic motion rather than uncorrelated hopping.

Time-correlation analyses quantify these dynamics (Fig. 4). The mean-squared displacement and self-intermediate scattering function reveal transient caging and stretched-exponential relaxation, marking cooperative slow dynamics. The four-point dynamic susceptibility $\chi_{4,C}\left(t\right)$ peaks near the freezing side of regime II, indicating growing spatial correlations, and collapses in the liquid phase, signifying a crossover from heterogeneous to homogeneous relaxation. The Stokes–Einstein ratios for carriers deviate strongly near the transition, confirming the emergence of cooperative transport beyond single-particle diffusion.

Spatially resolved mobility maps reveal pronounced dynamical heterogeneity near the sublattice-melting transition (Fig. 5*A*). Carriers segregate into coexisting mobile and immobile domains, with string-like cooperative displacements and correlated hopping events indicating collective, rather than single-particle, transport. Enhanced mobility preferentially appears near the boundaries of crystallized regions, where particles transiently occupy interstitial positions. The corresponding static snapshots (Fig. 5*B*) demonstrate a strong correspondence between local structural softness and mobility: Dynamically active regions coincide with locally compressed, liquid-like environments, while dynamically frozen regions remain structurally ordered. This spatial correlation parallels the behavior observed in glass-forming systems and implicates anharmonic lattice distortions as the microscopic origin of heterogeneous carrier dynamics near sublattice melting.

The Debye–Waller factor links these dynamical changes to local vibrations (Fig. 6). At high density, the host particles exhibit nearly harmonic behavior, whereas the carrier particles display increasingly nonlinear vibrational dynamics near the sublattice-melting regime, signaling enhanced anharmonicity. Reducing the packing fraction broadens regime III, softens the host lattice, and induces increasingly nonlinear fluctuations in both subsystems (Fig. 7), demonstrating that density controls the cooperativity of sublattice melting by reshaping the local energy landscape. Simulations of 3D α-AgI with Coulomb interactions (Fig. 8) reproduce qualitatively similar transport regimes, supporting the broader relevance of the mechanisms identified in the minimal model.

These insights suggest that sublattice melting can be engineered via density control, offering design principles for robust, high-conductivity solid electrolytes. The roles of dimensionality and carrier size in shaping transport behavior are examined in detail in *SI Appendix*.

## Discussions

While the present 2D NAP model captures the essential physics of sublattice melting, several directions remain for future exploration. Although the density-controlled sharp change in transport shows features often associated with phase transitions, its precise thermodynamic classification is subtle. We therefore use sublattice melting to denote the underlying mechanism–selective carrier delocalization driven by enhanced anharmonicity and spatially heterogeneous, string-like motion–rather than a strict phase-transition taxonomy. Importantly, this dynamical heterogeneity directly facilitates transport by generating interconnected high-mobility regions that enable cooperative carrier motion, shifting transport control from average energy barriers to the connectivity of these pathways. Within this framework, our results are consistent with prior studies reporting barrier reduction due to collective ion motion (62). Here, we show that this reduction arises from an entropic contribution, whereby collective dynamics enhance the migration entropy and thereby lower the effective activation barrier governing transport. A detailed decomposition of energetic and entropic contributions is provided in *SI Appendix*. While string-like cooperative dynamics and non-Arrhenius ionic transport have been reported in more realistic models (63), we show within our minimal NAP model that such behaviors arise from a unified mechanism in which anharmonic lattice fluctuations induce spatially heterogeneous dynamics near the onset of sublattice melting. In the high-temperature superionic state, carrier motion is constrained by bottlenecks imposed by the host lattice but remains dynamically homogeneous and liquidlike. Near the onset of sublattice melting, however, the dynamics become strongly heterogeneous, with transient coexistence of temporarily localized and liquidlike carrier regions, reminiscent of cooperative motion in glass-forming systems. This heterogeneity is progressively suppressed as the system evolves into the fully developed superionic regime. Although the model is formulated in two dimensions for simplicity and visualization, the observed dynamical behavior is not restricted to dimensionality or strict long-range translational order. Our chemistry-neutral minimal NAP model isolates the essential dynamical mechanism while omitting explicit long-range electrostatics and polarizability. Incorporating these effects is a natural extension and is expected to shift quantitative thresholds without altering the qualitative mechanism. Indeed, corresponding 3D simulations of α-AgI reproduce qualitatively similar diffusivity crossovers and transport regimes within the superionic phase, supporting the generality of the underlying dynamical behavior. To further clarify the microscopic origin of the transport regimes, we analyze the carrier dynamics of the corresponding 3D NAP system in *SI Appendix*. These simulations reproduce the same qualitative sequence of transport crossovers observed in two dimensions and further demonstrate that carrier size strongly influences the dominant high-temperature transport mechanism. In particular, comparatively larger carriers exhibit an additional activated regime associated with the onset of host-lattice melting, indicating a separation between carrier-controlled and host-controlled transport processes. Together, these results support the generality of the underlying dynamical picture across dimensionality and interaction regimes. To rigorously validate the microscopic mechanism proposed here, it is crucial to connect this coarse-grained framework with first-principles descriptions.

Experimentally, our findings offer clear guidelines for materials design. In real systems, the effective density–or equivalently, the lattice volume or chemical pressure–can be tuned through compositional substitution, strain, or external pressure; these controls correspond to effective lattice expansion and defect engineering that modulate free volume and local lattice stiffness. We predict that reducing the effective density broadens the sublattice-melting regime and enhances carrier anharmonicity, implying that low-density or expanded-lattice phases may exhibit sublattice melting at comparatively lower temperatures. Neutron and X-ray scattering measurements provide access to carrier diffusion, intermediate scattering functions, and Debye–Waller factors, enabling characterization of relaxation dynamics, cooperative motion, and the predicted crossover from harmonic to anharmonic behavior. Raman and quasielastic neutron spectroscopy can further probe phonon softening and correlated ionic motion for quantitative comparison with simulations. Signatures of dynamical heterogeneity may also be inferred from deviations from the Stokes–Einstein relation, obtained by combining diffusion measurements with spectroscopic relaxation times. Importantly, this framework provides a direct correspondence between microscopic dynamical processes–such as anharmonic fluctuations and cooperative carrier motion–and experimentally measurable transport properties, including ionic diffusivity and non-Arrhenius behavior, thereby providing a physically grounded interpretation of superionic conduction across a broad class of materials.

To further assess the quantitative relevance of the minimal NAP model beyond qualitative phenomenology, we compared the carrier diffusivity obtained at packing fraction ϕ=0.70 with experimental diffusion data for β-alumina in the Arrhenius regime associated with sublattice melting (23) (see *SI Appendix* for details). Although β-alumina contains distinct crystallographic diffusion sites (e.g., BR and a-BR) and associated energetic heterogeneity, our NAP model assumes equivalent sites and therefore isolates the underlying collective transport mechanism rather than reproducing material-specific microscopic details. By collapsing the Arrhenius regimes of the simulated and experimental diffusivity curves, we obtain an effective activation scale $\epsilon /k_{B}=1,264.3\;K$ and a transport prefactor $a^{2}/\tau_{0}=2.05\times 10^{-5}\;{\mathrm{cm}}^{2}\;s^{-1}$. These results further support the robustness of the underlying transport mechanism captured by the NAP model. Details of the fitting procedure, scaling, and physical interpretation of these parameters are provided in *SI Appendix*.

In conclusion, this study provides a quantitative and conceptually transparent framework that bridges phenomenological modeling with an atomistic understanding of sublattice melting. Unlike conventional mean-field or single-ion hopping descriptions–which treat ionic transport as independent motion in a static potential–our results reveal that fast ion conduction arises from collective, anharmonic, and density-dependent dynamics constrained by the crystalline host, offering a microscopic foundation for interpreting nonlinear transport behaviors observed in superionic conductors. Combining first-principles simulations with controlled experiments will be essential to verify the universality of these mechanisms and translate them into design principles for next-generation solid electrolytes.

## Materials and Methods

Molecular dynamics (MD) simulation codes were developed in C++ (2D and 3D NAP model) and LAMMPS (3D α-AgI model) (64), and postprocessing, analysis, and figure generation were carried out using Python scripts with Matplotlib (65) and OVITO (66).

### Two-Dimensional Nonadditive Potential (NAP) Model: Interactions and Simulation Protocol.

To reproduce the essential physics of sublattice melting within a minimal and chemically neutral framework, we impose distinct interaction types on the two species. The host particles interact through short-range steric repulsion, forming a mechanically rigid crystalline framework, whereas the carrier particles experience a much softer, effectively long-range Wigner-type interaction that promotes collective delocalization within the host lattice. This asymmetry in interaction range and stiffness naturally yields widely separated melting temperatures for the two sublattices, enabling the emergence of a selective carrier-melting phase in which the carrier sublattice becomes fluidlike while the host lattice remains ordered. We refer to this minimal, chemically neutral framework as the nonadditive potential (NAP) model.

To place our approach in context, prior studies of superionic conductors have shown that ion transport and sublattice-selective melting can emerge from relatively simple interaction schemes. In tunnel-type solids, a balance of Coulombic, polarization, and short-range repulsive forces governs the off-axis migration pathways and activation barriers of mobile ions, producing size-selective mobility (61, 67). More broadly, these studies demonstrate that coarse-grained molecular dynamics with simplified but physically motivated interactions can reproduce essential features of ion-conducting phases. Our NAP model follows this spirit but adopts an even more generalized, chemically neutral formulation, retaining the core interplay between host confinement and carrier delocalization while avoiding system-specific assumptions.

To this end, we consider a two-dimensional binary mixture comprising a total of N=512 particles, with equal numbers of host ($N_{H}=256$) and carrier ($N_{C}=256$) species. The moderate system size is intentionally chosen to suppress long-wavelength fluctuations associated with the Mermin–Wagner theorem in two dimensions, while preserving the intrinsic transport mechanisms of interest (47–49). As shown in Fig. 1*B*, the initial configuration of the binary mixture is constructed in a hexagonal packing arrangement, where the host particles define the underlying lattice and the carriers are distributed within the interstitial sites. The interparticle interactions are modeled using the Weeks-Chandler-Andersen (WCA) potential, a truncated and shifted form of the Lennard-Jones potential, as detailed in the following.

For a pair of particles i and j separated by a distance $r_{\mathrm{ij}}=\left|r_{i}-r_{j}\right|$, the interaction potential is given by $U=\sum_{\left\langle i,j\right\rangle }U_{\mathrm{ij}}\left(r_{\mathrm{ij}}\right)$ where,

[1] $$\begin{array}{c} U_{\mathrm{ij}}\left(r_{\mathrm{ij}}\right)=\left\{\begin{array}{cc} 4\epsilon_{\mathrm{ij}}\left[{\left(\frac{\sigma_{\mathrm{ij}}}{r_{\mathrm{ij}}}\right)}^{12}-{\left(\frac{\sigma_{\mathrm{ij}}}{r_{\mathrm{ij}}}\right)}^{6}\right]+\epsilon_{\mathrm{ij}}, & r_{\mathrm{ij}}\leq r_{c},\\ 0, & r_{\mathrm{ij}}>r_{c}, \end{array}\right. \end{array}$$

where the notation ⟨i,j⟩ denotes a sum over all distinct particle pairs (i≠j), with each pair counted only once. $\sigma_{\mathrm{ij}}$ is the effective particle diameter for the interacting pair and $\epsilon_{\mathrm{ij}}$ sets the energy scale. The cutoff distance $r_{c}$ = $2^{1/6}\sigma_{\mathrm{ij}}$ ensures that the potential is purely repulsive. In our simulation, we use $\sigma_{H}$, $\epsilon_{\mathrm{HH}}$, $m_{H}$, and $\sqrt{m_{H}\sigma_{H}^{2}/\epsilon_{\mathrm{HH}}}$ as units of length, energy, mass, and time, respectively. Temperature is expressed in reduced units as $T^{*}=k_{B}T/\epsilon_{\mathrm{HH}}$, where $\epsilon_{\mathrm{HH}}$ sets the characteristic energy scale of the system. A correspondence with real temperatures is established by matching the Arrhenius behavior of the simulated diffusivity to experimental data (see *SI Appendix*).

The interaction parameters are symmetric, i.e., $\sigma_{\mathrm{ij}}=\sigma_{\mathrm{ji}}$ and $\epsilon_{\mathrm{ij}}=\epsilon_{\mathrm{ji}}$, and the corresponding values of $\left(\sigma_{\mathrm{ij}},\epsilon_{\mathrm{ij}}\right)$ for all particle pair types are summarized in Table 1 (see Fig. 1*A* for detailed visualization). The host–host (HH) and carrier–host (CH) interactions are defined with a common energy scale $\epsilon_{\mathrm{ij}}=1.0$, whereas the carrier–carrier (CC) interaction is deliberately chosen to be much weaker, $\epsilon_{\mathrm{CC}}=10^{-3}$. This parameterization suppresses direct mutual exclusion between carriers and promotes collective dynamics within the confining host lattice, consistent with earlier coarse-grained descriptions of superionic transport based on long-range correlations rather than explicit short-range repulsion (6, 61). In contrast to those works, however, we do not introduce explicit charge-specific Coulomb interactions. Instead, we employ a nonadditive, long-range interaction in a minimal form, designed to capture the essential collective effects of carrier motion while remaining chemically neutral.

**Table 1.** Model parameters used in the 2D NAP model

$N_{C}:N_{H}$	$m_{C}$	$m_{H}$	$\epsilon_{\mathrm{CC}}$	$\epsilon_{\mathrm{HH}}$	$\epsilon_{\mathrm{CH}}$	$\sigma_{C}$	$\sigma_{H}$	$\sigma_{\mathrm{CH}}$
1:1	1.0	1.0	0.001	1.0	1.0	0.154	1.0	0.577

In this minimal framework, explicit electrostatic and polarization effects are neglected to isolate the roles of interaction asymmetry and lattice softness in sublattice melting and carrier dynamics. While such effects may alter quantitative scales (e.g., transition temperatures and transport coefficients), the qualitative features remain robust, as confirmed by simulations with more realistic interaction potentials (see below).

Accordingly, our two-dimensional NAP model captures key features of superionic behavior, including selective sublattice melting and strongly heterogeneous carrier dynamics, indicating that these phenomena arise primarily from collective effects governed by lattice softness and long-range correlations among mobile carriers.

The lattice spacing $a_{L-\mathrm{space}}$, effective carrier size $\sigma_{\mathrm{CC}}$, and the corresponding box dimensions $\left(L_{x},L_{y}\right)$ for the two-dimensional system at different area packing fractions ϕ are summarized in Table 2.

**Table 2.** Simulation box parameters at different area packing fractions *ϕ*

ϕ	$a_{L-\mathrm{space}}$	$\sigma_{\mathrm{CC}}$	$L_{x}$	$L_{y}$
0.85	1.0440	1.0440	16.7040	14.4661
0.80	1.0773	1.0773	17.2362	14.9270
0.75	1.1126	1.1126	17.8016	15.4166
0.70	1.1516	1.1516	18.4256	15.9570
0.65	1.1951	1.1951	19.1216	16.5598

We employed both underdamped Brownian dynamics (BD) and Newtonian molecular dynamics (MD) to evolve the binary mixture. During the initial equilibration stage, the system was evolved under underdamped Langevin dynamics, $m_{i}\frac{dv_{i}}{dt}=-\zeta v_{i}-\frac{\partial U}{\partial r_{i}}+\eta_{i}\left(t\right)$, where ζ is the damping coefficient and $\eta_{i}\left(t\right)$ is a Gaussian white noise term with zero mean: $\langle \eta_{i}\left(t\right)\rangle =0$ and the variance $\left\langle \eta_{i}\left(t\right)⊗\eta_{j}\left(t^{\prime }\right)\right\rangle =2k_{B}T\zeta \;\delta_{\mathrm{ij}}\delta \left(t-t^{\prime }\right)1$ due to fluctuation–dissipation theorem and 1 denotes the unit matrix. BD simulations were propagated for $2\times 10^{6}$ steps with timestep $\Delta t_{\mathrm{BD}}=0.001$, corresponding to a total equilibration time $t_{\mathrm{BD}}=2\times 10^{3}$. After equilibration, the system was switched to microcanonical molecular dynamics (NVE ensemble) to follow the Hamiltonian evolution. MD simulations were performed for $1\times 10^{8}$ steps with the time step $\Delta t_{\mathrm{MD}}=0.0001$, corresponding to a total production run length $t_{\mathrm{MD}}=1\times 10^{4}$.

In both stages, the system of N particles was propagated for sufficiently long times to ensure equilibration and statistical averaging. Brownian dynamics ensured relaxation into equilibrium configurations, whereas subsequent NVE molecular dynamics enabled the study of intrinsic dynamical processes without thermostatting.

### Radial Distribution Function.

The pair distribution function (PDF) of particles of type α∈{C,H} (carrier or host) is defined (68) as $g_{\alpha \alpha }\left(r\right)=\frac{1}{\rho_{\alpha }N_{\alpha }}\langle \sum_{j\neq k,\;j,k\in \alpha }\delta \left(r-r_{j}+r_{k}\right)\rangle ,$ where $\rho_{\alpha }=N_{\alpha }/\left(L_{x}L_{y}\right)$ is the number density of particle of species α and $N_{\alpha }$ is the number of particles of species α. The radial distribution function (RDF), $g_{\alpha \alpha }\left(r\right)$, was obtained by averaging the PDF over all angular directions. This function quantifies the local structural correlations within each particle species and provides a measure of the characteristic interparticle spacing and short/long-range order.

### Mean-Squared Displacement.

The mean-squared displacement (MSD) of particles of type α∈{C,H} (carrier or host) was computed as (69) $\left\langle \Delta r_{\alpha }{\left(t\right)}^{2}\right\rangle =\frac{1}{N_{\alpha }}\sum_{j\in \alpha }\langle [r_{j}\left(t+t_{0}\right)-r_{j}\left(t_{0}\right){]}^{2}\rangle .$ At long times, the MSD exhibits diffusive behavior, from which the self-diffusion coefficient $D_{\alpha }$ is determined as $\left\langle \Delta r_{\alpha }{\left(t\right)}^{2}\right\rangle ∼4D_{\alpha }t.$ This analysis was conducted separately for the carrier and host species, allowing a direct comparison of their respective mobilities and of the temperature dependence of their transport dynamics.

### Self-Intermediate Scattering Function.

The self-intermediate scattering function of particles of type α∈{C,H} (carrier or host) was calculated as (68, 70) $F_{s,\alpha }\left(q,t\right)=\frac{1}{N_{\alpha }}\sum_{j\in \alpha }\langle \exp\left[iq\cdot (r_{j}\left(t+t_{0}\right)-r_{j}\left(t_{0}\right))\right]\rangle ,$ where q is the wave vector, whose magnitude was chosen to correspond to the characteristic length scale of the host particles, i.e., near the first peak of the static structure factor. Relaxation dynamics was characterized by monitoring the temporal decay of $F_{s,\alpha }\left(q,t\right)$. In the following, we restrict the analysis to the carrier species. The structural relaxation time for carriers $\tau_{\alpha ,C}$ was determined by fitting $F_{s,C}\left(q,t\right)$ to the Kohlrausch–Williams–Watts (KWW) function, $F_{s,C}\left(q,t\right)∼A\exp\left\{-{\left[t/\tau_{\alpha ,C}\right]}^{\beta }\right\}$, where A and β are the fitting parameters that represent the amplitude and stretching exponent, respectively. This analysis was performed to directly probe the microscopic relaxation and temperature-dependent dynamics of the carriers.

### Four-Point Dynamic Susceptibility.

To quantify dynamical heterogeneity consistent with our analysis of $F_{s,\alpha }\left(q,t\right)$, we evaluated the four-point dynamic susceptibility (52) for particles of type α∈{C,H} (carrier or host) using the self-overlap order parameter. The single-particle overlap is defined as $w_{j}^{\left(\alpha \right)}\left(t_{0}+t,t_{0}\right)\equiv \Theta (a-|r_{j}\left(t_{0}+t\right)-r_{j}\left(t_{0}\right)|)$, where Θ(·) is the Heaviside step function and a is a microscopic cutoff that sets the cage scale. Unless otherwise noted, we set $a=a_{\text{L-space}}/\left(2\sqrt{3}\right)$, a geometric threshold corresponding to half the nearest-neighbor spacing in the host lattice, ensuring that the overlap function probes cage-breaking displacements on the host lattice length scale.

The species-resolved (self) overlap is $Q_{\alpha }\left(t\right)=\sum_{j\in \alpha }w_{j}^{\left(\alpha \right)}\left(t_{0}+t,t_{0}\right)$, with averages taken over $t_{0}$ (multiple time origins) and independent trajectories. The four-point dynamic susceptibility is then obtained from the variance of $Q_{\alpha }\left(t\right)$ as $\chi_{4,\alpha }\left(t\right)=\frac{1}{N_{\alpha }}\left(\left\langle Q_{\alpha }{\left(t\right)}^{2}\right\rangle -{\left\langle Q_{\alpha }\left(t\right)\right\rangle }^{2}\right)$. In reporting $\chi_{4,\alpha }\left(t\right)$, we highlight a characteristic time: the peak time $\tau_{\chi_{4}}$ that maximizes $\chi_{4,\alpha }\left(t\right)$. All quantities were computed separately for carrier particles only to compare the species-resolved growth of dynamic correlations and their temperature dependence.

### Lindemann Index and Debye–Waller Factor.

To quantify species-resolved local relative fluctuations in particle positions, we computed the two-dimensional Lindemann index (32, 60, 71–76) $\gamma_{L,\alpha }\left(t\right)$ for each component α∈{C,H} (carrier or host). Following the definition of the relative displacement between particles j and k, $u_{\mathrm{jk}}\left(t\right)=[r_{j}\left(t\right)-r_{k}\left(t\right)]-[r_{j}\left(0\right)-r_{k}\left(0\right)]$, the Lindemann index for each species is defined as $\gamma_{L,\alpha }\left(t\right)=\frac{1}{N_{\alpha }}\sum_{j\in \alpha }\frac{1}{n_{j}}\sum_{k\in \alpha ,\;n.n.(j)}\langle |u_{\mathrm{jk}}\left(t\right){|}^{2}\rangle /a_{L-\mathrm{space}}^{2}.$ Here, n.n.(j) denotes the set of first-nearest-neighbor host particles surrounding particle j. The integer $n_{j}$ is the coordination number of particle j, determined from the first minimum of the host–host radial distribution function. The Lindemann index $\gamma_{L,\alpha }\left(t\right)$ thus measures the relative amplitude of the local vibrational motion of α particles with respect to their nearest-neighbor distances on the host-particle length scale. This function was computed independently for carrier and host particles, allowing direct comparison of their local structural stability and the temperature dependence of their relative fluctuations.

The Debye–Waller factor quantifies the a mplitude of vibrational motion in the caged regime and is closely related to the plateau value of the Lindemann index. For each species α∈{C,H} (carrier or host), we define $u_{\alpha }^{2}=\gamma_{L,\alpha }\left(t_{p}\right),$ where $\gamma_{L,\alpha }\left(t_{p}\right)$ denotes the value taken at the characteristic turning time $t_{p}$ between the ballistic and diffusive regimes. This time marks the cage-confinement timescale at which both the mean-squared displacement and $\gamma_{L,\alpha }\left(t\right)$ exhibit a transient plateau. For host particles (α=H), $u_{H}^{2}$ corresponds directly to the plateau height of $\gamma_{L,H}\left(t\right)$. For carrier particles (α=C), the plateau is less pronounced due to stronger anharmonicity; accordingly, $u_{C}^{2}$ is evaluated at the same $t_{p}$ to represent their effective vibrational amplitude. This definition enables a consistent comparison of Debye–Waller factors between the two species and provides a microscopic proxy for the local stiffness of their respective environments. If $u_{\alpha }^{2}\propto T$ holds, the dynamics in that regime can be regarded as effectively harmonic.

### Three-Dimensional α-AgI (FUH) Model: Interactions and Simulation Protocol.

To demonstrate the generality of our coarse-grained Coulombic framework, we perform molecular dynamics simulations of a 3D ionic conductor inspired by α-AgI, following the Fukumoto–Ueda–Hiwatari (FUH) model (61).

The interaction potential between ions i and j separated by a distance $r_{\mathrm{ij}}$ is given by

[2] $$\begin{array}{c} V_{\mathrm{ij}}\left(r_{\mathrm{ij}}\right)=\epsilon {\left(\frac{\sigma_{i}+\sigma_{j}}{r_{\mathrm{ij}}}\right)}^{7}+\frac{C\;q_{i}q_{j}}{r_{\mathrm{ij}}}, \end{array}$$

which combines short-range repulsion with long-range Coulomb interactions. Here $q_{i}=z_{i}f$ denotes the dimensionless effective charge of ion i, where $z_{i}$ is the valency (e.g., $z_{\mathrm{Ag}}=+1$, $z_{I}=-1$) and f is the ionicity parameter. The Coulomb interaction is characterized by the prefactor $C=e^{2}/\left(4\pi \varepsilon_{0}\right)=14.3996\;\mathrm{eV}$ Å, which sets the electrostatic energy scale. In the simulations, this prefactor is absorbed into the chosen unit system (eV for energy and Å for length). This formulation is equivalent to the conventional expression $z_{i}z_{j}{\left(fe\right)}^{2}/r_{\mathrm{ij}}$ used in the original α-AgI (FUH) model (61). The long-range interactions are evaluated using Ewald summation under periodic boundary conditions.

The parameters used in the simulations are as follows. The energy scale is ϵ=0.177eV with ionicity f=1.0. The ionic radii are $\sigma_{I}=2.2$ Å for iodide and $\sigma_{\mathrm{Ag}}=0.63$ Å for silver, giving effective pair diameters $\sigma_{I-I}=4.4$ Å, $\sigma_{\mathrm{Ag}-\mathrm{Ag}}=1.26$ Å, and $\sigma_{I-\mathrm{Ag}}=2.83$ Å. A cutoff radius of $r_{c}=12.0$ Å is used. The lattice constant is a=5.08 Å, and the system size corresponds to N=256 particles. The simulations are performed with a time step Δt=0.0093 ps and a thermostat damping parameter τ=0.1 ps, over a total of $10^{6}$ steps. The system consists of Ag^+^ and I^−^ ions arranged in a body-centered cubic lattice with mobile silver ions occupying interstitial sites. Simulations are performed in the *NVT* ensemble using a Nosé–Hoover thermostat and integrated via the velocity-Verlet algorithm.

This model retains the essential ingredients of our two-dimensional system–long-range interactions, size asymmetry, and mobile charge carriers–demonstrating that the observed dynamical behavior is robust across dimensions.

## Supplementary Material

Appendix 01 (PDF)

Movie S1. **Unconstrained carrier transport in the fully molten regime (*T* = 7.00)**. To visualize carrier transport within the host sublattice, we generated trajectory movies for a 2D NAP model system at area packing fraction *ϕ* = 0.85. Three movies were prepared to illustrate carrier dynamics across different thermal regimes. Particle identities are color-coded to track individual trajectories to assess the carrier motion throughout the system. Movie S1 (S14) corresponds to a high-temperature state (*T* = 7.00), well above the melting temperature. The trajectories are shown over a time window [*t*_0_, *t*_0_ + 40 *τ_α_*], where *τ_α_* denotes the structural relaxation time at *T* = 7.00. In this regime, both the carrier and host sublattices are fully molten, eliminating geometric constraints imposed by the host. As a result, no bottleneck effects are observed, and the system exhibits liquid-like behavior characterized by homogeneous carrier motion and uniform spatial distribution.

Movie S2. **Sublattice melting with liquid-like carriers and rigid host lattice (*T* = 2.50)**. Movie S2 (S15) shows particle trajectories at temperature *T* = 2.50, analogous to Movie S1. The system is initialized from a well-relaxed, spatially homogeneous configuration, indicating that it is in a steady state. Despite the relatively high temperature, the dynamics reveal a clear separation between carrier and host degrees of freedom. While the carrier particles exhibit liquid-like motion and undergo long-range transport, the host lattice remains largely immobile over the observation window. As a result, the carrier trajectories develop pronounced bottleneck structures around the fixed host sites, leading to persistent low-occupancy regions coinciding with the host lattice positions. This coexistence of a mobile carrier sublattice with a structurally stable host lattice provides direct dynamical evidence of sublattice melting: the carrier sublattice is fully melted and percolates through the system, whereas the host lattice retains its positional order. The resulting heterogeneous flow pathways highlight the constrained nature of carrier transport imposed by the rigid host framework, even at elevated temperatures.

## Data Availability

Anonymized numerical data set data have been deposited in Zenodo (77). All other data are included in the manuscript and/or *SI Appendix*.
