# Supplementary material for: Probing anharmonic and heterogeneous carrier dynamics across sublattice melting in a minimal model superionic conductor
Source: Proc Natl Acad Sci U S A. 2026 Jul 7;123(28):e2605867123. doi: 10.1073/pnas.2605867123 (PMC13367812; doi:10.1073/pnas.2605867123)
Supplement: Supplementary file 1 — Appendix 01 (PDF) [file pnas.2605867123.sapp.pdf]

Supporting Information for

Probing Anharmonic and Heterogeneous Carrier Dynamics Across Sublattice Melting in a Minimal Model Superionic Conductor

Sucharita Niyogi, Takenobu Nakamura, Genki Kobayashi, Yasunobu Ando, and Takeshi Kawasaki

Takeshi Kawasaki
E-mail: kawasaki.takeshi.d3c@osaka-u.ac.jp
Sucharita Niyogi
E-mail: niyogi.sucharita.d3c@osaka-u.ac.jp

This PDF file includes:

Supporting text
Figs. S1 to S15
Legends for Movies S1 to S2
SI References

Other supporting materials for this manuscript include the following:

Movies S1 to S2

Purpose and Structure of the SI Appendix

In the main text, we introduced a minimal two-dimensional binary model that isolates the microscopic origin of fast ion transport by disentangling sublattice melting, anharmonicity, and dynamical heterogeneity in a chemically agnostic framework. A rigid host lattice coexists with a soft carrier sublattice, producing a broad regime in which carriers delocalize while the host remains crystalline. Structural, dynamical, and time-correlation analyses demonstrate that fast transport in this regime arises from collective, anharmonic carrier motion rather than independent hopping.

While our study establishes the central phenomenology and its physical interpretation, several aspects merit further clarification and quantitative support. The Supporting Information addresses these points in detail and provides additional structural and dynamical diagnostics used to characterize sublattice order, collective carrier motion, and deviations from harmonic behavior across the density-controlled crossover discussed in the main text. We first discuss issues related to the quantitative correspondence between simulation observables and experimental measures, providing additional context for interpreting diffusivity and structural metrics. We then examine the dimensional robustness of the sublattice melting and high-temperature transport behavior by extending the analysis to three-dimensional (3D) systems. The dependence of sublattice melting on carrier size in three dimensions is analyzed next, highlighting how steric confinement and migration barriers reshape the transport landscape. We further present a detailed analysis of the temperature evolution of the radial distribution function for the two-dimensional system at the packing fraction $\phi = 0.85$, clarifying the structural signatures associated with carrier delocalization. Finally, we investigate hyperuniformity and static density fluctuations through the structure factor, providing additional insight into long-wavelength correlations in the selectively melted state. In addition, we provide further analysis of lattice anharmonicity through the Lindemann index and Debye–Waller factor, together with a systematic examination of the density dependence of carrier dynamics and host-lattice fluctuations in the two-dimensional (2D) non-additive potential (NAP) model. We also clarify the geometric origin of the honeycomb-like carrier trajectories observed in the main text and discuss the connection between collective hopping, migration entropy, and effective activation barriers within a transition-state framework. The effects of partial carrier occupancy and the temperature dependence of spatial dynamical heterogeneity are further examined to assess the robustness of the collective transport mechanism identified in this work.

Together, these supporting analyses reinforce the conclusions of the main results, clarify the limits of the minimal model we proposed, and outline directions for connecting sublattice-melting physics to broader classes of superionic and glassy materials.

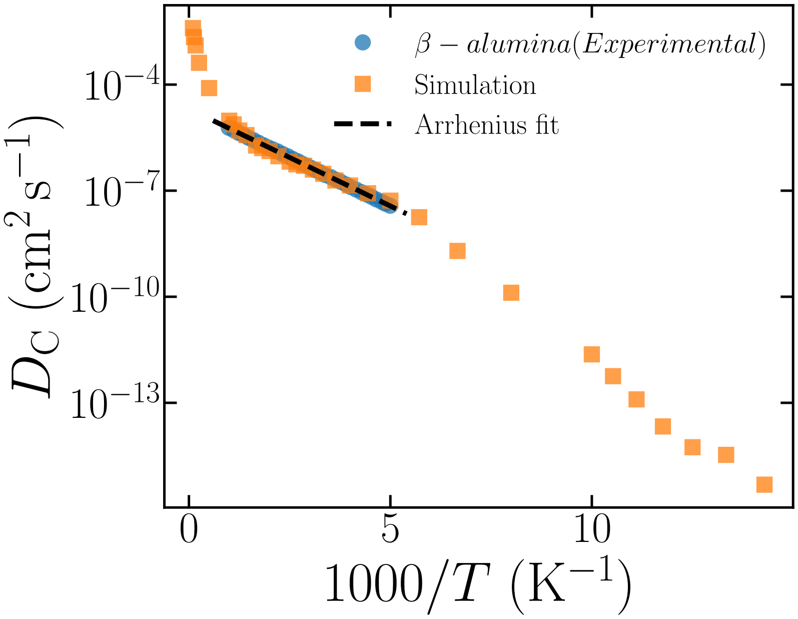

Fig. S1. Collapse of simulated carrier diffusivity (orange squares) onto experimental β-alumina data (blue circles) in the Arrhenius regime. Simulation data are rescaled to experimental units. The black dotted line shows the common Arrhenius fit, demonstrating quantitative agreement between simulation and experiment.

A. Quantitative mapping between simulation and experiment. To assess the quantitative validity of our minimal numerical model, we directly compared the carrier diffusivity obtained from two-dimensional simulations at packing fraction $\phi = 0.70$ with experimental diffusion data for β-alumina (1), as shown in Fig. S1. The comparison was performed in the Arrhenius regime associated with sublattice melting, where both simulation and experiment exhibit approximately activated transport.

Our choice of β-alumina as a reference system is motivated by clear structural and dynamical parallels with our minimal NAP model. In both cases, mobile ions are constrained to a partially occupied sublattice, resulting in collective transport on a reduced-dimensional network (1). In particular, the effective dimensionality and site occupancy in our two-dimensional simulations closely resemble those of Na-β-alumina conduction planes, where ionic motion is largely confined within well-defined

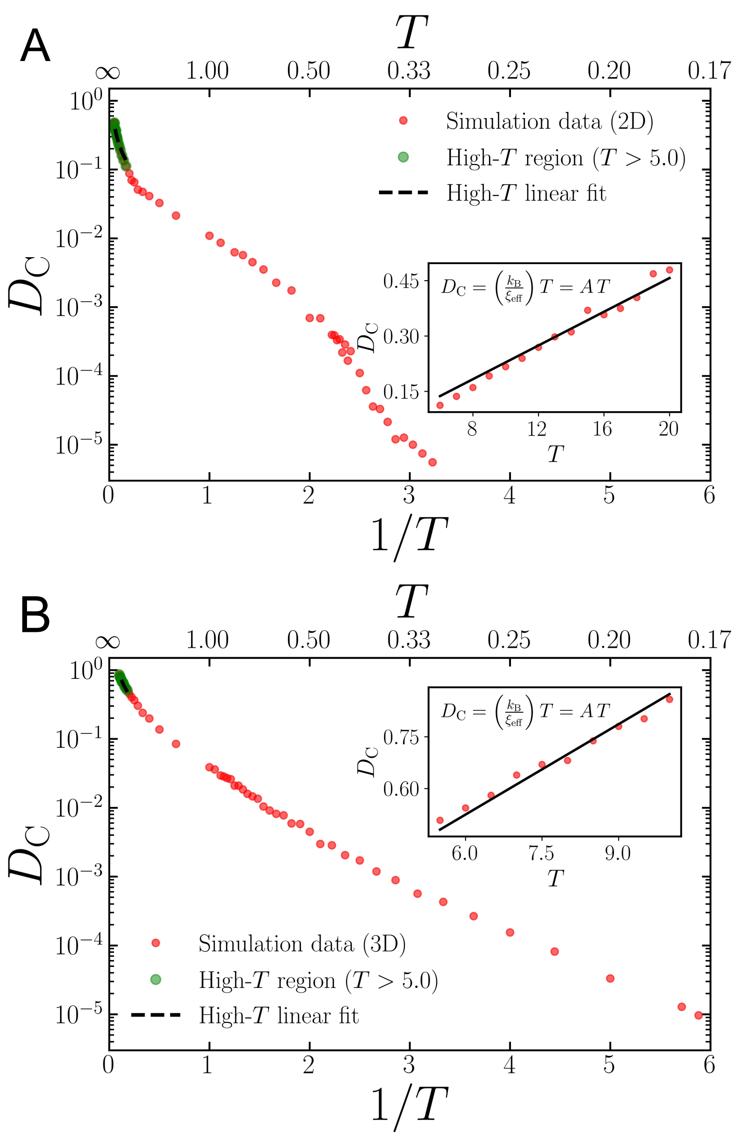

Fig. S2. High-temperature transport of small carriers in two and three dimensions. (*A*) Two-dimensional system at packing fraction $\phi = 0.85$ (similar to Fig. 2*A* of the main text). (*B*) Three-dimensional system at volume fraction $\phi_{\mathrm{volume}} = 0.62$. In both panels, the main plots show the carrier diffusivity D_{C} as a function of inverse temperature $1/T$. A distinct high-temperature regime emerges in which the diffusivity exhibits weak curvature in the Arrhenius representation. The shaded (green) symbols highlight the high-temperature region used for further analysis. Insets show D plotted directly as a function of T for this high-temperature regime, together with linear fits of the form $D_{\mathrm{C}} = (k_{\mathrm{B}}/\xi_{\mathrm{eff}})\,T = A\,T$. The excellent linearity observed in both two and three dimensions confirms the validity of the Einstein relation in this regime, indicating a crossover to a kinetic, weakly constrained transport mechanism at elevated temperatures.

layers.

At the microscopic level, Na-β-alumina exhibits multiple site types (e.g., BR and a-BR) with distinct local environments, which can introduce energetic heterogeneity and domain-like structures (1). In contrast, our model assumes energetically equivalent sites, thereby isolating the essential transport physics from material-specific complexity. From this perspective, β-alumina serves as a representative sublattice-mediated ionic conductor, and the comparison is intended not for material-specific reproduction but to demonstrate that the collective, anharmonic transport mechanism identified in the simulations is consistent with experimental behavior.

Now, the experimental diffusivity follows an Arrhenius form,

$$D_{\mathrm{exp}}(T) = \frac{a^2}{\tau_0}\exp\left(-\frac{\epsilon}{k_{\mathrm{B}}T}\right), \qquad [1]$$

where ϵ is an effective activation energy and a^2/τ_0 is a transport prefactor with dimensions of diffusivity. In the simulations, temperature and diffusivity are expressed in reduced units. To relate the two, we performed a direct collapse of the Arrhenius portions of the inverse-temperature plots by matching both the slope and intercept over the interval corresponding to the sublattice-melting regime.

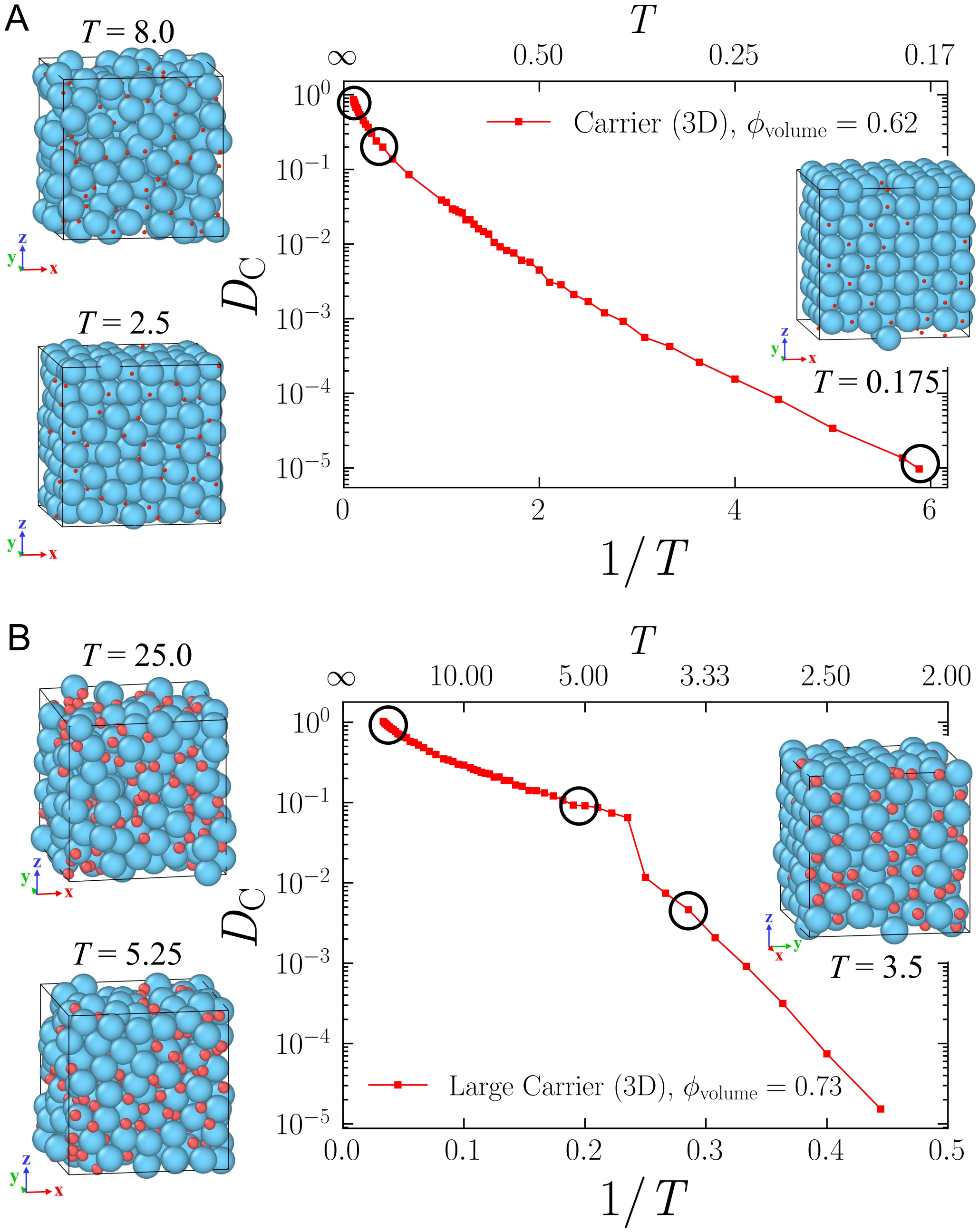

Fig. S3. Carrier diffusivity D_{C} as a function of inverse temperature $1/T$ in the three-dimensional system for two carrier sizes. (*A*) Small carriers show high mobility and a broad sublattice-melting regime while the host lattice remains crystalline (same as Fig. S2*B*). Snapshots at $T = 0.175$ reveal liquid-like carrier motion within an ordered host, at $T = 2.5$ a dynamically disordered, percolating carrier network with intact host order, and at $T = 8.0$ complete melting of both sublattices. (*B*) Larger carriers exhibit a sharp onset of diffusion near host-lattice melting. Configurations at $T = 3.5$ indicate carrier sublattice melting, at $T = 5.25$ signatures of partial host-lattice destabilization, and at $T = 25.0$ full melting of the crystal. Black circles mark the corresponding temperatures.

This procedure yields an effective activation scale $\frac{\epsilon}{k_{\mathrm{B}}} = 1264.3$ K, in quantitative agreement with reported activation energies for fast-ion conduction in β-alumina (1). The vertical alignment of the Arrhenius plots further determines the transport prefactor $\frac{a^2}{\tau_0} = 2.05 \times 10^{-5}\ \mathrm{cm}^2\,\mathrm{s}^{-1}$.

Importantly, only the ratio a^2/τ_0 is fixed by the collapse; separating the characteristic length scale a and the microscopic time scale τ_0 would require additional assumptions about single-particle hopping dynamics that lie outside the scope of the present minimal, chemically agnostic model. We therefore interpret a^2/τ_0 as an effective transport scale encoding collective, anharmonic carrier motion rather than independent activated hops.

This quantitative agreement demonstrates that the emergence of fast ion transport across sublattice melting in the simulations reproduces not only the qualitative phenomenology, but also the correct experimental activation scale and transport prefactor,

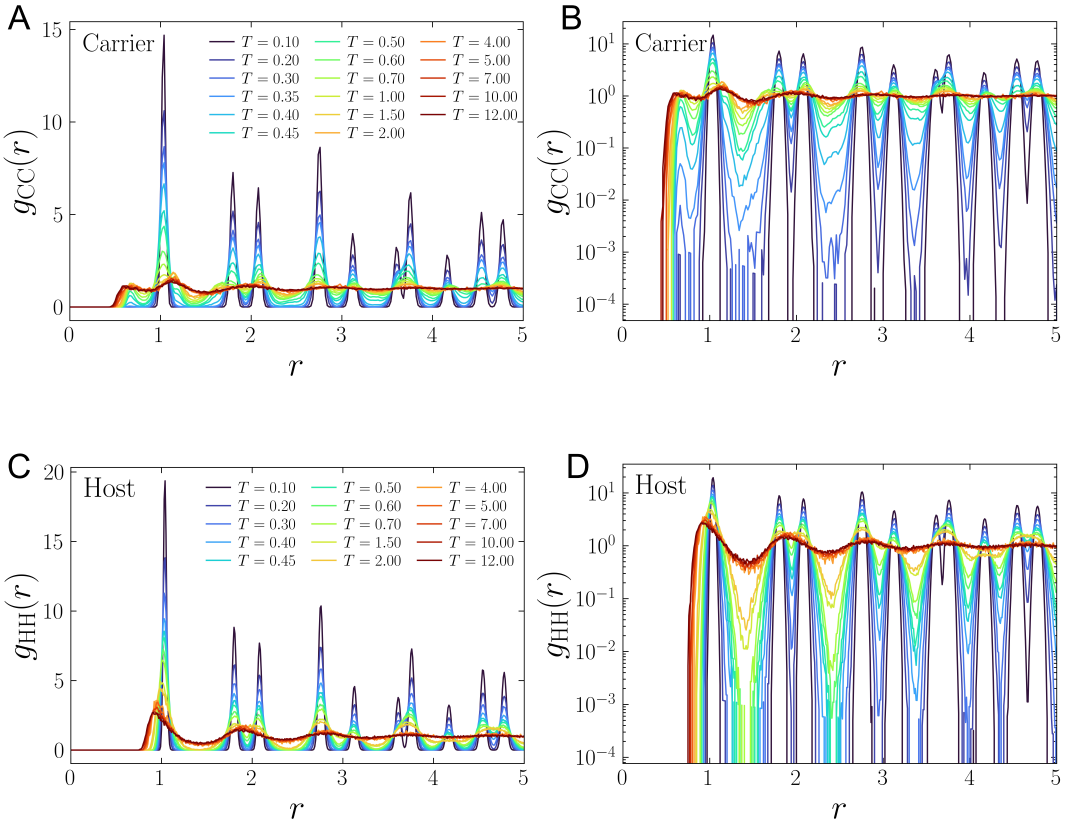

Fig. S4. Temperature dependence of the radial distribution function $g_{\alpha\alpha}(r)$ for the two-dimensional system at packing fraction $\phi = 0.85$. (*A–B*) Carrier (α: C) distribution $g_{\mathrm{CC}}(r)$ at low and elevated temperatures, showing sharp crystalline peaks at low T and the emergence of an excess shoulder at higher T. (*C–D*) Host (α: H) distribution $g_{\mathrm{HH}}(r)$, which remains largely ordered until complete melting, with negligible excess peaks or shifts. The logarithmic representation (*B, D*) highlights subtle features of the distribution.

supporting the relevance of collective and anharmonic mechanisms as the microscopic origin of superionic transport.

B. Dimensional robustness and high-temperature transport behavior.

Three-dimensional NAP model: simulation protocol. To test the robustness of the observed behavior against dimensionality, we performed complementary 3D molecular dynamics simulations based on the same physical design principles as the 2D model. Initially the interaction hierarchy, size asymmetry, and mass ratios between host and carrier species were kept identical as the 2D NAP model (see "Materials and Methods" in main text), isolating dimensionality as the only difference.

The initial configuration was constructed on an FCC-like lattice with alternating host and carrier particles in all three directions. The simulation box comprised a periodic array of $4 \times 4 \times 4$ unit cells ($N \simeq 512$ particles with equal species populations). Host particles had diameter $\sigma_{\mathrm{H}} = 1.0$, carriers $\sigma_{\mathrm{C}} = 0.154$, and both species had equal masses. The lattice spacing $a_{\text{L-space}} = 1.5$ corresponds to a volume packing fraction $\phi_{\text{volume}} = 0.6228$, yielding a cubic box of size $L = 6.0$ with periodic boundary conditions.

Interactions followed the same scheme as in two dimensions: stiff, purely repulsive host–host and host–carrier interactions stabilized the host crystal, while softened carrier–carrier interactions promoted correlated carrier motion. After equilibration, microcanonical production runs were performed.

High-temperature transport of carriers in 2D and 3D systems. We first characterize the high-temperature transport regime and its dependence on dimensionality for the small-carrier system (carrier size $\sigma_{\mathrm{C}} = 0.154$), where steric constraints are minimal. Figure S2 compares the carrier diffusivity D as a function of inverse temperature $1/T$ in two dimensions at packing fraction $\phi = 0.85$ (Fig. S2*A*) and in three dimensions at volume fraction $\phi_{\text{volume}} = 0.62$ (Fig. S2*B*), using identical interaction parameters and carrier size (see "Materials and Methods" in main text).

In both cases, the diffusivity initially follows an activated, Arrhenius-like behavior at low temperatures, reflecting carrier motion constrained by the crystalline host lattice. Upon increasing temperature, the system enters an intermediate sublattice-melting regime in which carrier mobility increases rapidly while the host lattice remains largely intact. In three dimensions, the additional configurational freedom smooths this low-temperature crossover, such that the deeply activated regime evident in two dimensions is compressed to lower temperatures and not fully resolved within the accessible simulation window. Following

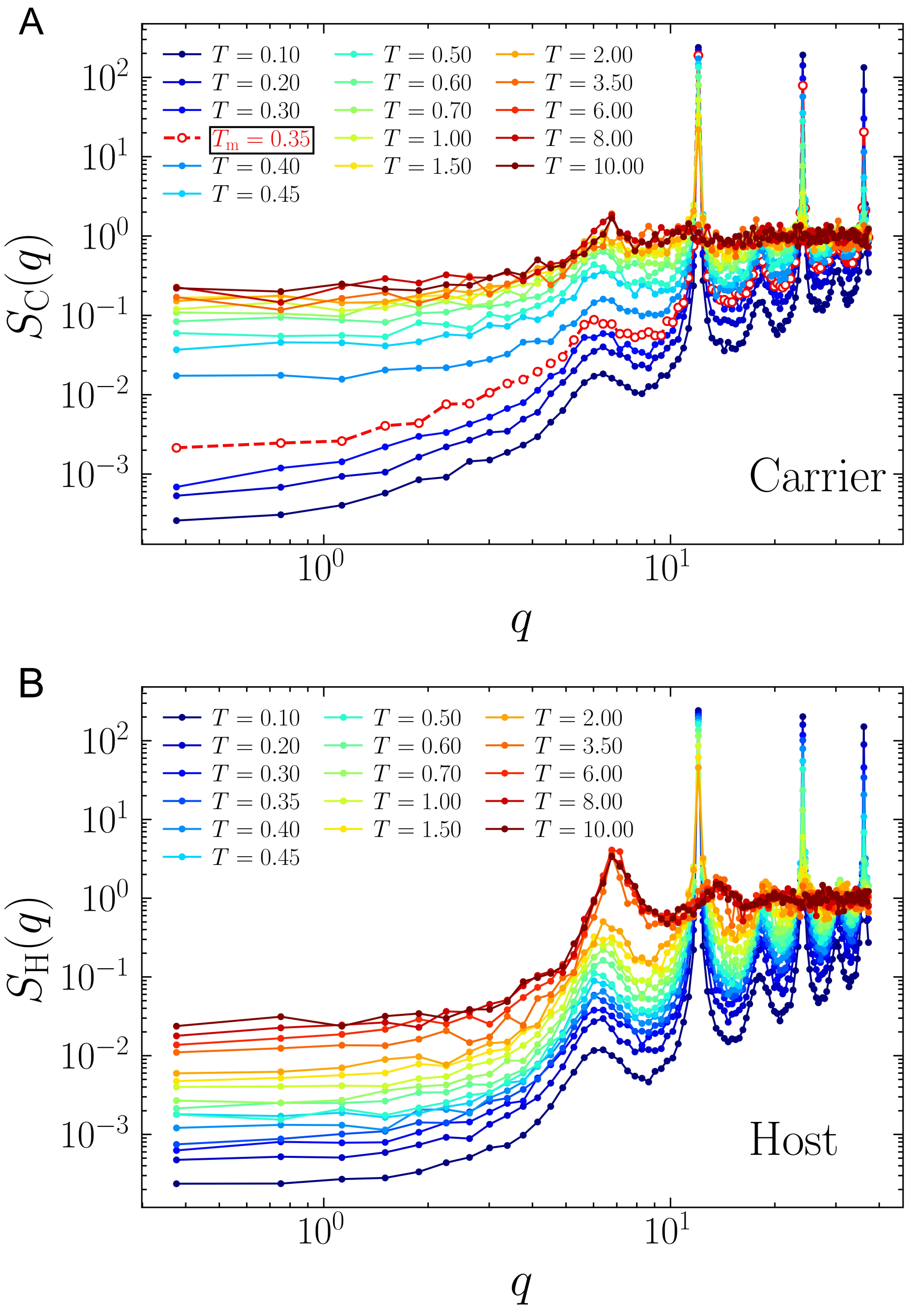

Fig. S5. Static structure factor $S_\alpha(q)$ for the two-dimensional system at packing fraction $\phi = 0.85$. (*A*) Carrier particles and (*B*) host particles at representative temperatures across the sublattice-melting regime. Carriers exhibit strong small-q suppression at low temperatures, which disappears upon melting ($T > T_{\mathrm{m}}$), whereas the host lattice retains pronounced Bragg-like order over a broader temperature range.

full melting of the host lattice, the diffusivity does not saturate, but instead crosses over into a distinct high-temperature transport regime characterized by a nearly linear increase of diffusivity D with temperature T.

This crossover is made explicit in the insets of Fig. S2, where D is plotted directly as a function of T. In this high temperature regime, the diffusivity is well described by $D = (k_{\mathrm{B}}/\xi_{\mathrm{eff}})T = AT$, consistent with the Einstein relation for kinetic, weakly constrained transport. Linear fits yield $A = k_{\mathrm{B}}/\xi_{\mathrm{eff}} = 2.29 \times 10^{-2}$ for the two-dimensional system and $A = k_{\mathrm{B}}/\xi_{\mathrm{eff}} = 8.74 \times 10^{-2}$ for the three-dimensional system. The robustness of this linear scaling across dimensionality demonstrates that, once structural constraints are sufficiently relaxed, carrier motion is governed primarily by thermal energy rather than by activation over persistent barriers. Notably, the same sequence of transport regimes—activated at low T, an intermediate sublattice-melting crossover, and the high-temperature linear $D \propto T$ behavior—is observed in three dimensions, confirming that the phenomenology is not an artifact of two-dimensionality.

This behavior stands in clear contrast to that observed for large carriers (discussed in the next section), where the diffusivity develops a weakly temperature-dependent, saturation-like regime at high temperatures. In those systems, transport remains dominated by residual activation barriers even after host melting, leading to an approximately temperature-independent diffusivity. The absence of such saturation in the small-carrier system highlights a qualitative change in the dominant transport mechanism, underscoring the role of carrier size in determining whether the high-temperature dynamics are barrier-limited or kinetic in nature.

C. Carrier-size dependence of sublattice melting in three dimensions. We examine how carrier size controls the emergence and character of sublattice melting in three dimensions by comparing transport and structural signatures for different carrier diameters at fixed lattice spacing $a_{\mathrm{L-space}} = 1.5$.

Figure S3*A* shows the carrier diffusivity D as a function of inverse temperature $1/T$ for small carriers with $\sigma_{\mathrm{C}} = 0.154$. The diffusivity exhibits a broad intermediate temperature regime in which D remains finite and only weakly temperature dependent over an extended interval. This behavior signals a wide sublattice-melting window, during which carriers progressively delocalize and form a dynamically connected network while the host lattice retains long-range crystalline order. The persistence of finite diffusivity throughout this regime indicates that carrier motion is not governed by a single dominant activation barrier, but instead reflects a distribution of low-energy migration pathways enabled by the size mismatch between carriers and the host lattice. Notably, this phenomenology closely mirrors that observed in two dimensions (see previous section), demonstrating that the emergence of an extended sublattice-melting regime for small carriers is robust to dimensionality.

Representative configurations at selected temperatures within this regime, marked by black circles in Fig. S3*A*, provide direct structural support for this interpretation. At $T = 0.175$, carriers already display liquid-like mobility despite a fully ordered host lattice, indicating that sublattice melting sets in deep in the low-temperature regime. At $T = 2.5$, the host lattice remains intact while the carrier subsystem forms a dynamically disordered, percolating structure. Only at $T = 8.0$ do both sublattices lose structural order, marking complete melting of the crystal.

In contrast, for larger carriers with $\sigma_{\mathrm{C}} = 0.42$ (Fig. S3*B*), strong steric confinement within the host lattice leads to substantially enhanced effective migration barriers. Carrier mobility remains strongly suppressed until the host lattice begins to lose structural integrity, producing a sharp onset of diffusion near the host melting temperature, followed by a weakly temperature-dependent, saturation-like diffusivity at higher temperatures. Notably, this combination of an abrupt activation of mobility and subsequent diffusivity saturation closely resembles the transport signatures reported in several superionic conductors (2). Structural analysis confirms that this apparent saturation-like behavior corresponds to a regime of *partial host melting*, in which ordered and disordered host regions coexist and continue to impose geometric constraints on carrier motion (see the snapshot at $T = 5.25$). This behavior can be rationalized by an activated form, $D \sim \exp(-\Delta E/k_{\mathrm{B}}T)$, which at sufficiently high temperatures may be expanded as $\exp(-\Delta E/k_{\mathrm{B}}T) \simeq 1 - \Delta E/k_{\mathrm{B}}T$, yielding only a weak residual temperature dependence. Frequent collisions and persistent geometric constraints within the partially disordered matrix further suppress the growth of diffusivity, leading to the observed saturation-like behavior.

Upon further heating, the diffusivity exhibits an additional sharp increase, signaling complete melting of the host lattice (see the snapshots at $T = 25.0$ showing the complete melting scenario) and the disappearance of persistent steric constraints. In this high-temperature limit, the system crosses over to a liquid-like transport regime in which carrier motion becomes weakly correlated and predominantly governed by thermal energy. Consistent with previous observations, the diffusivity in this regime follows an Einstein-like scaling $D \propto k_{\mathrm{B}}T$, indicating that transport is no longer controlled by activation over geometric barriers but by kinetic motion in a fully disordered environment. We note that this ultimate high-temperature crossover, while clearly resolved in simulations, is expected to be difficult to access experimentally. The temperatures required to fully eliminate residual steric constraints typically exceed those attainable in stable solid-state or superionic materials, where thermal decomposition or chemical degradation intervene. As a result, experimental measurements often probe only the intermediate, weakly temperature-dependent regime, which may appear as an apparent saturation of diffusivity.

Together, these results demonstrate that the carrier size acts as a key control parameter governing both the width of the sublattice-melting regime and the dominant transport mechanism at high temperatures. Small carriers transition to a kinetic, Einstein-like transport regime once structural constraints are lifted, whereas large carriers remain effectively barrier-limited even in the disordered state. Similar qualitative behavior can also be induced in two dimensions by tuning model parameters.

D. Static property: Radial distribution function (RDF) $g_{\alpha\alpha}(r)$ of the system. In this section, we next analyse the temperature evolution of the radial distribution function (see "Materials and Methods" in the main text) for carriers $g_{\mathrm{CC}}(r)$ and hosts $g_{\mathrm{HH}}(r)$ separately. As shown in Fig. S4*A*, the carrier distribution at low temperatures exhibits sharp, periodic peaks, characteristic of crystalline order. Upon heating, a distinct shoulder emerges on the left of the first peak and gradually intensifies, accompanied by peak broadening and shifts near the sublattice-melting regime. The appearance of this excess feature reflects activated single-carrier hopping events associated with Frenkel-type disorder (3) (see Fig. S4*B* for its enhanced visibility on a logarithmic scale). With increasing temperature, these structural signatures indicate that the carrier sublattice progressively loses positional order and acquires liquid-like characteristics, while the host lattice remains largely intact. In contrast, the host distribution (Fig. S4*C*–*D*) shows neither the excess peak nor noticeable shifts until its complete melting. This clear disparity highlights the heterogeneous nature of the system, where mobile carriers undergo an early transition to a fluid-like state within an otherwise ordered host framework.

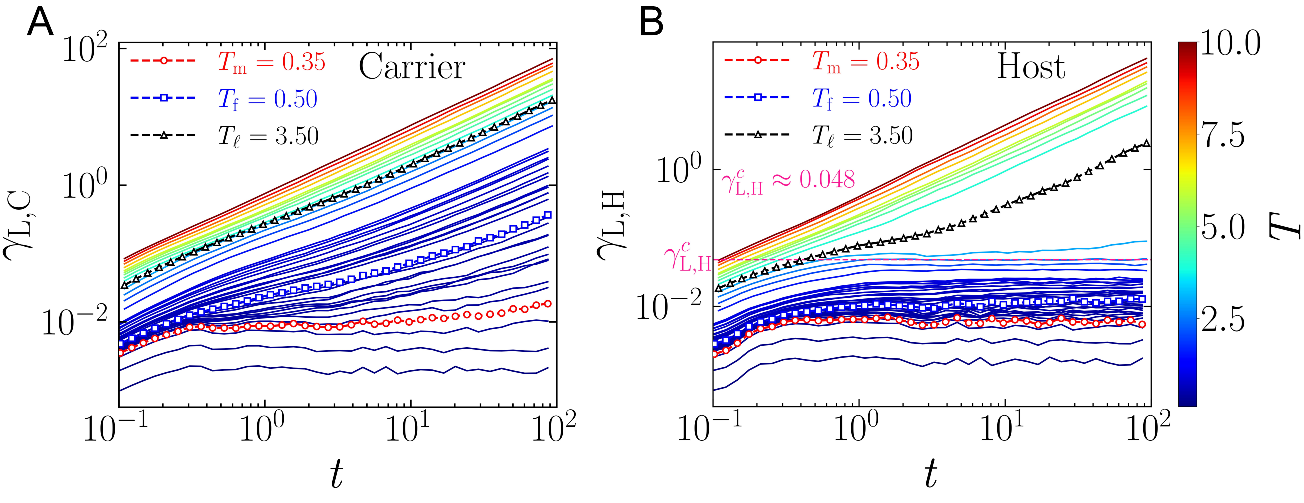

Fig. S6. Lindemann index $\gamma_{L,\alpha}$ and effect of anharmonicity: (*A–B*) Time evolution of the Lindemann index for carriers and hosts at packing fraction $\phi = 0.85$. For the host lattice (*A*), the Lindemann index shows a clear signature of lattice melting, while for the carriers (*B*) the growth is purely driven by anharmonic fluctuations rather than a melting transition.

E. Hyperuniformity: Structure factor. We analyze the structure factor

$$S_\alpha(q) = \frac{1}{N_\alpha} \left\langle \rho_\alpha(\mathbf{q})\, \rho_\alpha(-\mathbf{q}) \right\rangle \tag{2}$$

for both the carrier and host lattices at different temperatures (see Fig. S5), where N_α is the number of particles of species α, $\rho_\alpha(\mathbf{q}) = \sum_{j\in\alpha} e^{i\mathbf{q}\cdot\mathbf{r}_j}$ is the Fourier component of the density, and $\langle\cdots\rangle$ denotes an ensemble average. At low temperatures, the carrier particles exhibit strong hyperuniformity, reflected in a vanishing $S_\alpha(q \to 0)$, which indicates suppressed long-wavelength density fluctuations and persistent crystalline correlations (4). Such hyperuniformity is also characteristic of ordered systems such as Wigner crystals, consistent with the quasi-crystalline arrangement of the carrier sublattice observed here. As the system approaches the sublattice melting regime, $S_\alpha(q)$ for carriers develops pronounced fluctuations and the small-q suppression disappears, signaling the breakdown of hyperuniformity and the loss of long-range crystalline correlations. Microscopically, the loss of hyperuniformity is localized near the temperature T_{m} (see the highlighted red circles in Fig. S5*A*). For $T < T_{\mathrm{m}}$, the carrier sublattice remains hyperuniform, consistent with a Wigner-crystal–like state in which long-wavelength density fluctuations are suppressed. Near T_{m}, carriers increasingly occupy heterogeneous, interstitial configurations, resulting in enhanced long-wavelength density fluctuations and the breakdown of hyperuniformity. This real-space heterogeneity is reflected in pronounced spatial variations of local carrier environments. This transition reflects the fragility of the carrier sublattice: once thermal fluctuations overcome the ordering, the carriers rapidly become disordered. Even after complete melting, the carrier $S_{\mathrm{C}}(q)$ exhibits only moderate peak heights, consistent with a fluid-like state lacking strong residual correlations.

In contrast, the host lattice maintains structural order over a broader temperature range. Although hyperuniformity is weaker for hosts at low temperatures, a sharp Bragg-like peak emerges and grows significantly after the host lattice completes melting, reflecting the development of long-range positional correlations as the host particles reorganize. These observations highlight the asymmetric melting dynamics between carriers and hosts, and the sensitivity of hyperuniformity to sublattice destabilization.

F. Lindemann Index Calculation. The evolution of the Lindemann index (see "Materials and Methods" of the main text) reveals a pronounced enhancement of anharmonicity as the system approaches the sublattice melting point (Fig. S6). Figs. S6*A,B* show the time evolution of the Lindemann index, $\gamma_{\mathrm{L},\alpha}(t)$, for the host and carrier particles at packing fraction $\phi = 0.85$. For the host lattice, $\gamma_{\mathrm{L,H}}(t)$ exhibits a well-defined plateau reflecting harmonic vibrations within a stable crystal, followed by a rapid increase once $\gamma_{\mathrm{L,H}}(t)$ exceeds the critical value $\gamma^{c}_{\mathrm{L,H}} \simeq 0.048$, consistent with the conventional Lindemann criterion for lattice melting and close to previously reported critical values $\gamma^{c}_{\mathrm{L}} \simeq 0.03$ for crystalline solids (5). In contrast, $\gamma_{\mathrm{L,C}}(t)$ for the carriers increases smoothly without a sharp transition, indicating that their fluctuations are governed by intrinsic anharmonic vibrations rather than by a structural instability.

G. Origin of honeycomb-like carrier trajectories. To clarify the origin of the honeycomb-like trajectories observed in Fig. 3 of the main text, we note that these patterns do not indicate hopping through host sites. Instead, they reflect the geometry of the interstitial network defined by the host lattice, together with the finite temporal resolution of trajectory sampling.

 Sucharita Niyogi, Takenobu Nakamura, Genki Kobayashi, Yasunobu Ando, and Takeshi Kawasaki

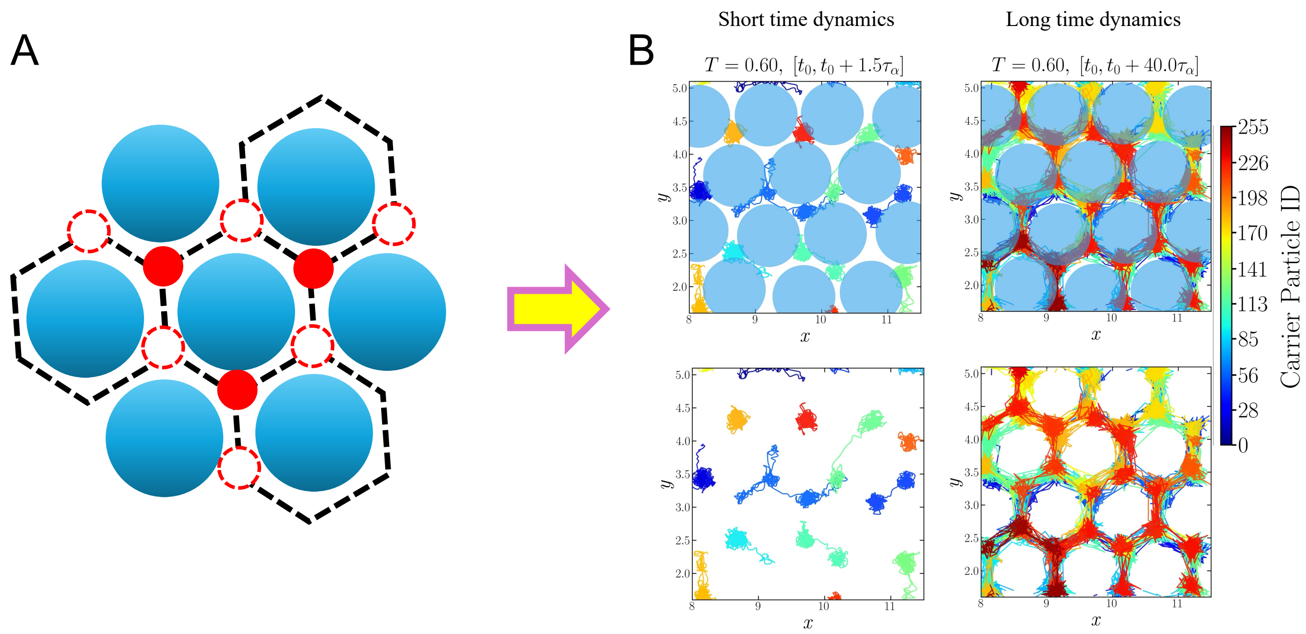

Fig. S7. Carrier trajectories and interstitial network structure: Trajectory of carrier particles forming hexagonal-like structures. (*A*) Schematic illustration: cyan particles represent host particles and red particles represent carriers. The dashed honeycomb network indicates the expected connectivity of interstitial pathways, and the dashed circular regions denote interstitial sites accessible to carriers. (*B*) Actual carrier trajectories overlaid on host particle positions. Colors of the trajectories correspond to particle IDs, following the same convention as in Fig. 3 in the main text. Importantly, carrier motion is confined to the interstitial network and does not pass through host particle cores.

As shown in Fig. S7*A*, the host particles form a hexagonal lattice. The interstitial voids define a connected network of accessible sites for the carriers, with pathways (indicated by black dotted lines) that are topologically equivalent to a honeycomb lattice. Carrier motion is therefore confined to these interstitial channels and naturally produces honeycomb-like trajectory patterns, while host cores remain excluded.

The apparent straight segments arise from the finite sampling interval. For example, in the case of $T = 0.60$, configurations are recorded every 500 steps (short-time) and 3900 steps (long-time), so intermediate positions are not resolved, leading to visual interpolation across excluded regions.

To clarify this point, Fig. S7*B* shows a zoomed-in view with host particles overlaid (upper panel) and carrier trajectories alone (lower panel). Host particles are shown at their reference positions; their thermal displacements are small and do not alter the connectivity of the interstitial network. These plots confirm that carriers remain confined to interstitial regions and do not pass through host sites.

H. Density dependence of lattice anharmonicity and carrier dynamics in our 2D NAP model. To elucidate the role of density in controlling lattice dynamics and carrier transport, we perform a systematic analysis across different packing fractions. We focus first on the Debye–Waller factor (DWF) of the host lattice as a measure of vibrational amplitude and lattice stiffness.

As shown in Fig. S8, the density dependence of the DWF reveals a clear change in behavior. At lower density ($\phi = 0.65$), the host exhibits an early deviation from linear temperature dependence as the system approaches the melting transition, indicating enhanced lattice softness and increased anharmonicity. In contrast, at higher density ($\phi = 0.85$), the DWF remains approximately linear over a wider temperature range, consistent with a more rigid lattice.

To further elucidate how this difference in lattice response affects carrier dynamics, we analyze spatial maps of particle mobility near the onset of sublattice melting (Fig. S9). At high density ($\phi = 0.85$), the dynamics are strongly heterogeneous and spatially localized, with pronounced string-like cooperative motion. These correlated displacements lead to the formation of extended immobile regions, reflecting constrained transport through a rigid environment. In contrast, at lower density ($\phi = 0.65$), the softer lattice supports more spatially extended mobility. Here, slow domains become broader while immobile regions shrink, resulting in more liquid-like and less spatially localized dynamics with weaker cooperative character.

In addition, we performed a structure factor analysis to quantify long-wavelength density fluctuations across densities (Fig. S10). At high density ($\phi = 0.85$), the carrier structure factor exhibits $S_{\rm C}(q) \to 0$ as $q \to 0$ at low temperatures, indicating a strong tendency toward hyperuniformity and suppressed large-scale density fluctuations. In contrast, at lower density ($\phi = 0.65$), $S_{\rm C}(q)$ approaches a finite constant in the same limit, demonstrating the absence of hyperuniformity and significantly enhanced density fluctuations.

These reciprocal-space results are fully consistent with the real-space observations: higher density enforces stronger collective constraints, leading to localized, string-like motion and pronounced dynamical heterogeneity, whereas lower density promotes broader, less correlated mobility and a more gradual crossover in dynamical behavior.

Importantly, these results show that density does not merely act as a static structural parameter, but serves as a control

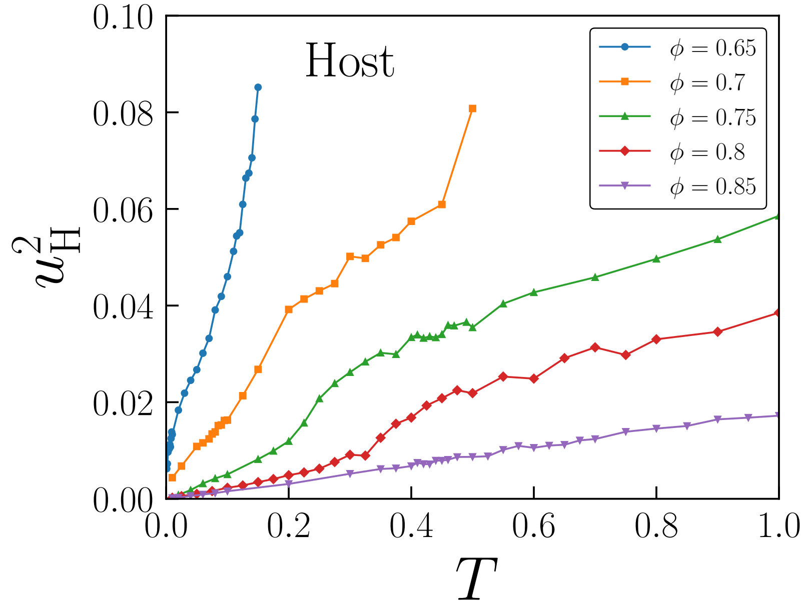

Fig. S8. Density dependence of the host Debye–Waller factor (DWF) as a function of temperature T. While the high-density case ($\phi = 0.85$) shows nearly linear (harmonic-like) behavior (same as Fig. 6 of the main text), lower-density systems display pronounced deviations from linearity, signaling the onset of anharmonic lattice dynamics associated with lattice softening.

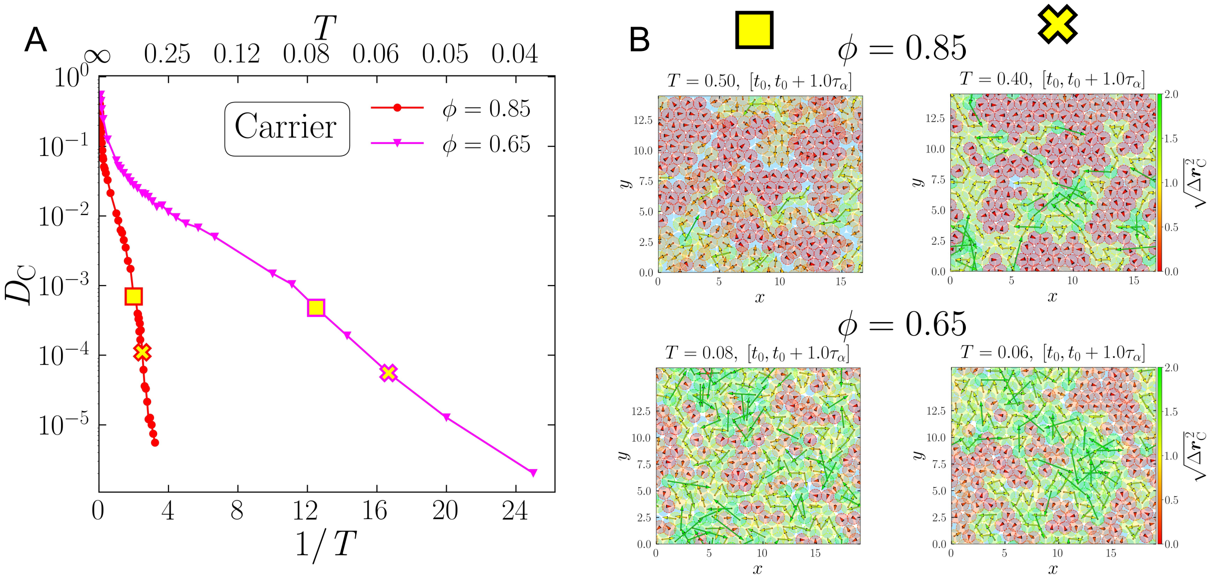

Fig. S9. Density dependence of carrier dynamics. (*A*) Carrier diffusivity (D_{C}) as a function of inverse temperature ($1/T$) for $\phi = 0.65$ and 0.85, highlighting the shift in the onset of sublattice melting. (*B*) Spatial maps of carrier displacements $\sqrt{\Delta \boldsymbol{r}_{\mathrm{C}}^2}$ over $[t_0, t_0 + \tau_\alpha]$ near the onset: for $\phi = 0.85$ ($T = 0.50, 0.40$), dynamics are strongly heterogeneous with localized, string-like cooperative motion; for $\phi = 0.65$ ($T = 0.08, 0.06$), mobility is more spatially extended with weaker heterogeneity due to increased lattice softness.

parameter for dynamical properties. In particular, changing density systematically modifies lattice stiffness, which in turn alters the energy landscape experienced by carriers, leading to changes in anharmonicity, cooperative dynamics, and the spatial structure of mobility. This establishes a direct mechanistic link between density and the dynamical quantities emphasized in this work—phonon anharmonicity, collective diffusion, and heterogeneous carrier dynamics—rather than a purely structural interpretation. In real materials, similar effects would manifest not through a single density parameter, but via changes in lattice volume, bonding strength, or local coordination environments induced by composition or external tuning. While such modifications inevitably involve changes in material chemistry, they effectively tune the same physical quantities identified in our model, namely lattice softness and dynamical heterogeneity, which govern transport behavior.

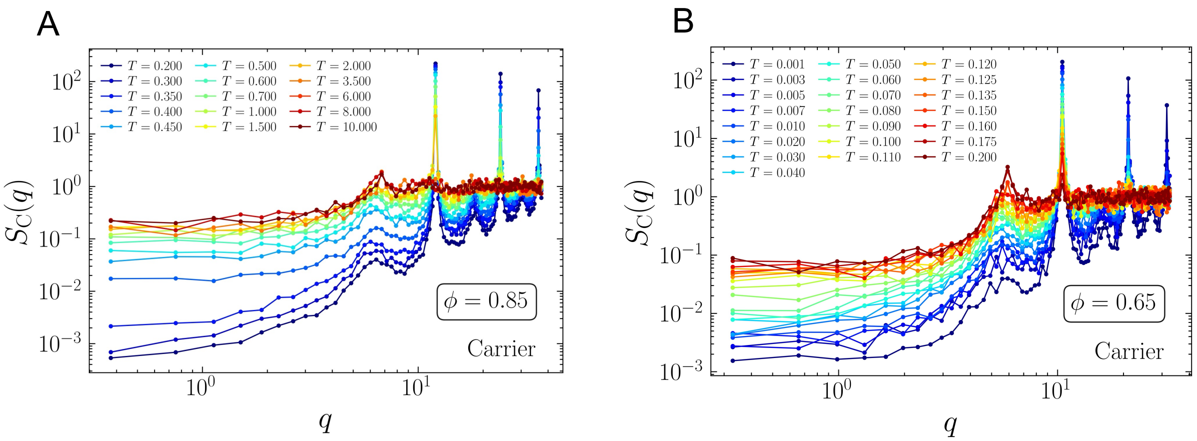

Fig. S10. Density dependence of the static structure factor $S_C(q)$ of the carrier particles at different temperatures T. (A) At higher density ($\phi = 0.85$), a pronounced suppression of $S_C(q)$ in the low-q regime at low T indicates strong hyperuniform behavior. (B) At lower density ($\phi = 0.65$), $S_C(q)$ remains finite at small q even at very low T, indicating the absence of hyperuniformity.

I. Connection between collective hopping, migration entropy, and effective activation barriers. To clarify the relationship between collective hopping dynamics and activation barriers, it is important to distinguish between the activation energy ΔE_a and the effective free-energy barrier ΔF governing ionic transport. Within a transition-state framework, the diffusivity can be expressed as (6, 7)

$$D \sim \exp\left(\frac{\Delta S_m}{k_B}\right) \exp\left(-\frac{\Delta E_a}{k_B T}\right) = \exp\left(-\frac{\Delta F}{k_B T}\right), \qquad [3]$$

where ΔS_m is the migration entropy, and

$$\Delta F = \Delta E_a - T\Delta S_m, \qquad [4]$$

is the free-energy barrier (effective activation energy). From the Boltzmann principle, the migration entropy can be interpreted as

$$\Delta S_m = k_B \ln\left(\frac{\Omega^\ddagger}{\Omega_0}\right), \qquad [5]$$

where $\Omega^\ddagger$ and Ω_0 denote the number of accessible configurations at the transition state and in the initial state, respectively.

As shown in Fig. S11, ΔS_m increases from C_{min} to C_{max} as the system evolves from regime I $\to$ II $\to$ III (sublattice melting). Here, $C_{min} \sim O(1)$ corresponds to a limited number of localized hopping carriers, whereas $C_{max} \sim O(N)$ reflects a regime in which a macroscopic number of carriers contribute to transport. Since the energetic contribution ΔE_a remains approximately constant due to the persistence of the host lattice, the increase in ΔS_m leads to a reduction of the effective free-energy barrier ΔF, thereby explaining the observed temperature dependence of D.

On the basis of this framework, the behavior observed in our system (Fig. S11) can be understood as follows:

Region I: (low temperature): Transport is dominated by single-particle hopping. The number of accessible pathways is limited, leading to an approximately constant migration entropy ΔS_m. As a result, the diffusivity follows standard Arrhenius behavior.
Region II: (onset of sublattice melting): This regime is characterized by the emergence of collective hopping events. These cooperative motions significantly increase the number of accessible configurations, leading to a strong temperature dependence of ΔS_m. This entropic enhancement produces a deviation from Arrhenius behavior and gives rise to an apparent change in slope.
Region III: (fully developed superionic state): Although the migration entropy is substantially larger than in Region I, it becomes approximately temperature-independent due to the homogeneous availability of multiple diffusion pathways. Consequently, Arrhenius behavior is recovered, with a slope similar to Region I.
Region IV: Complete melting, including the host lattice. In this regime, ΔE_a differs from that in the crystalline host phase.

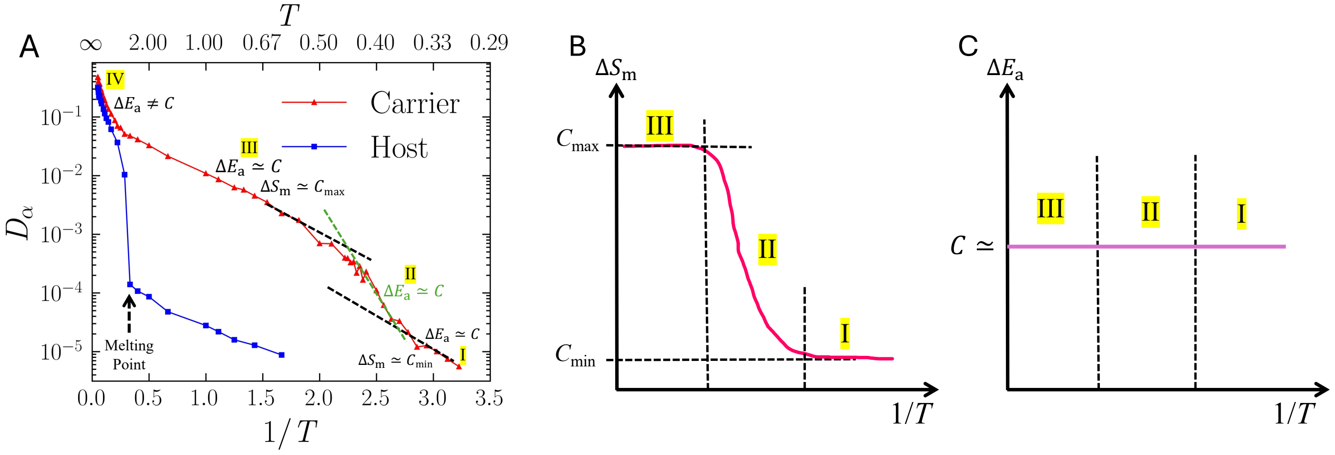

Fig. S11. Effective energy barrier of the system. (*A*) Arrhenius plot (D_α vs. $1/T$), analogous to Fig. 2*A* (of the main text), with annotations highlighting the behavior of the activation energy ΔE_a and migration entropy ΔS_m across temperature regimes. (*B*) Schematic illustration of the migration entropy ΔS_m in each regime, emphasizing its qualitative variation and its role in governing transport. (*C*) Activation energy ΔE_a as a function of $1/T$, showing an approximately constant behavior in regimes I–III, consistent with an intact host lattice.

J. Effect of partial occupancy on carrier dynamics in our NAP model. From a physical perspective, reducing the occupancy increases the availability of vacant sites (see Fig. S12), which can weaken the degree of cooperativity in hopping dynamics. This may shift the onset of collective dynamics to different temperatures or reduce the magnitude of the migration entropy enhancement. Nevertheless, the central mechanism identified in this work—namely, the increase of migration entropy ΔS_m due to the proliferation of accessible hopping pathways, leading to a reduction in the effective barrier ΔF—is expected to remain valid (see previous section as well). Thus, our framework generalizes from collective transport in dense systems to vacancy-mediated transport in dilute systems, providing a unified description across different occupancy regimes.

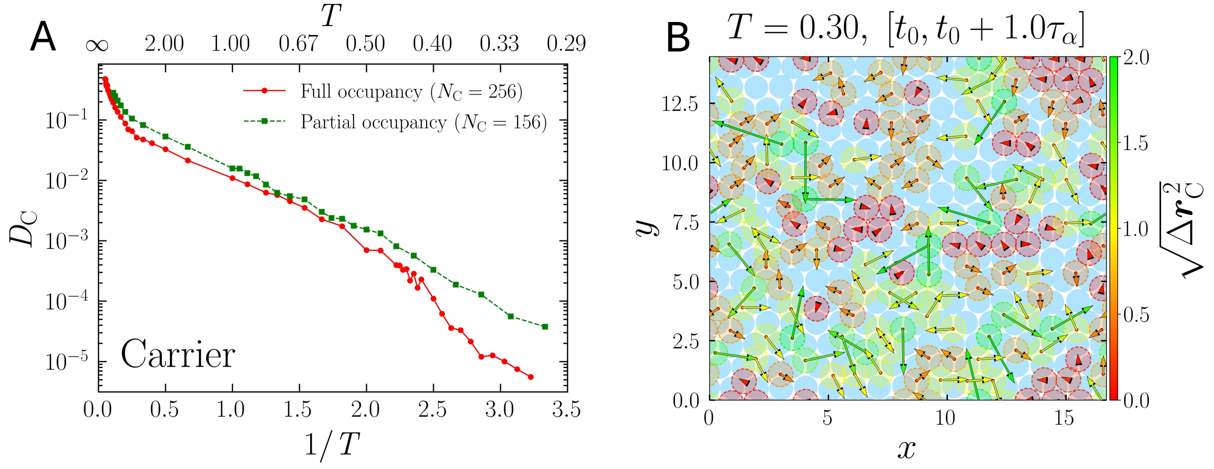

Fig. S12. Dynamics of partially occupied carrier systems. (*A*) Arrhenius plot of the diffusion coefficient for carriers (D_C) as a function of inverse temperature ($1/T$), comparing a fully occupied system (same as for our 2D NAP model for $\phi = 0.85$) with a partially occupied system. While both systems exhibit Arrhenius-like behavior at high temperatures, the partially occupied system shows higher diffusivity and weaker dynamical slowing at low temperatures due to reduced crowding and vacancy-assisted motion. (*B*) Spatial mobility map of the partially occupied carrier system at $T = 0.30$. Arrows indicate carrier displacements over the observation interval $[t_0,\ t_0 + \tau_\alpha]$. Unlike the fully occupied case, the dynamics do not exhibit pronounced string-like cooperative motion or strongly localized dynamical heterogeneity, indicating that transport is dominated primarily by spatially dispersed single-particle hopping events under partial occupancy.

To further examine this effect, we considered a reduced carrier concentration ($N_\mathrm{C} : N_\mathrm{H} = 156 : 256$) and compared the resulting dynamics with the fully occupied case ($N_\mathrm{C} : N_\mathrm{H} = 256 : 256$), as shown in Fig. S12*A*. At high temperatures, both systems exhibit comparable Arrhenius-like transport behavior. However, clear differences emerge in the low-temperature regime, where the partially occupied system maintains systematically higher diffusivity and exhibits a substantially weaker dynamical slowdown than the fully occupied case. In contrast to the pronounced crossover behavior observed for full occupancy, the partially occupied system displays an approximately single-Arrhenius temperature dependence over the investigated range.

To clarify the microscopic origin of this behavior, we analyzed the spatial mobility patterns in the low-temperature diffusive regime ($T = 0.30$), shown in Fig. S12*B*. Unlike the fully occupied system, the partially occupied state does not exhibit

pronounced string-like cooperative motion or strongly heterogeneous collective rearrangements. Instead, the dynamics are dominated by relatively isolated and spatially dispersed hopping events, indicating that diffusion primarily proceeds through vacancy-assisted single-particle motion. The suppression of collective hopping pathways under partial occupancy is therefore consistent with the disappearance of the crossover between cooperative and noncooperative transport regimes, leading to the observed single-Arrhenius behavior.

K. Temperature dependence of spatial dynamical heterogeneity. To directly visualize the evolution of dynamical heterogeneity, we examine spatial maps of particle mobility across different temperature regimes. Figure S13 shows the distribution of particle displacements over the interval $[t_0, t_0 + \tau_\alpha]$ at representative temperatures below and above the sublattice melting temperature T_{f}.

At low temperatures ($T < T_{\mathrm{f}}$), the system exhibits pronounced spatial heterogeneity, characterized by the coexistence of highly mobile regions and localized, quasi-crystalline domains. As the temperature increases toward T_{f}, these heterogeneous regions progressively diminish, indicating a reduction in spatio-temporal correlations in particle motion. Above the sublattice melting regime ($T > T_{\mathrm{f}}$), the dynamics become increasingly homogeneous, with particle mobility distributed more uniformly across the system.

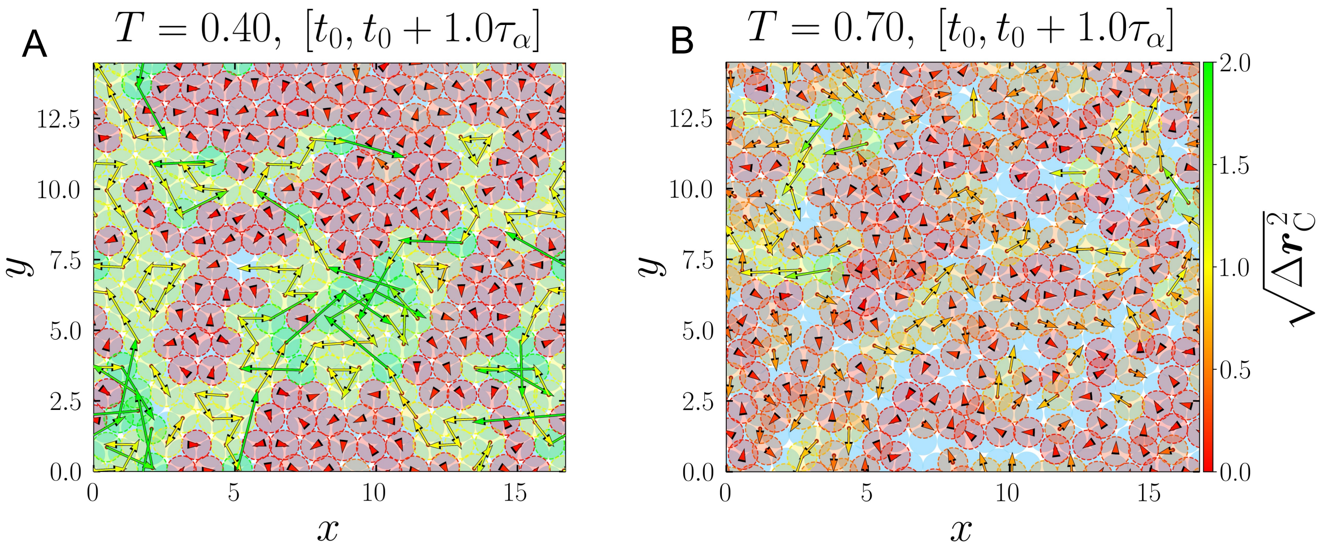

Fig. S13. Temperature evolution of spatial mobility maps at $\phi = 0.85$. (*A*) $T = 0.4$ ($T < T_{\mathrm{f}}$) and (*B*) $T = 0.7$ ($T > T_{\mathrm{f}}$). At low temperature, pronounced dynamical heterogeneity is observed through coexisting mobile and localized regions, which diminishes as the system transitions to a more homogeneous dynamical state at higher temperatures.

Movie S1. Unconstrained carrier transport in the fully molten regime ($T = 7.00$)

To visualize carrier transport within the host sublattice, we generated trajectory movies for a 2D NAP model system at area packing fraction $\phi = 0.85$. Three movies were prepared to illustrate carrier dynamics across different thermal regimes. Particle identities are color-coded to track individual trajectories to assess the carrier motion throughout the system. Movie S1 (S14) corresponds to a high-temperature state ($T = 7.00$), well above the melting temperature. The trajectories are shown over a time window $[t_0,\, t_0 + 40\,\tau_\alpha]$, where τ_α denotes the structural relaxation time at $T = 7.00$. In this regime, both the carrier and host sublattices are fully molten, eliminating geometric constraints imposed by the host. As a result, no bottleneck effects are observed, and the system exhibits liquid-like behavior characterized by homogeneous carrier motion and uniform spatial distribution.

Movie S2. Sublattice melting with liquid-like carriers and rigid host lattice ($T = 2.50$)

Movie S2 (S15) shows particle trajectories at temperature $T = 2.50$, analogous to Movie S1. The system is initialized from a well-relaxed, spatially homogeneous configuration, indicating that it is in a steady state.

Despite the relatively high temperature, the dynamics reveal a clear separation between carrier and host degrees of freedom. While the carrier particles exhibit liquid-like motion and undergo long-range transport, the host lattice remains largely immobile over the observation window. As a result, the carrier trajectories develop pronounced bottleneck structures around the fixed host sites, leading to persistent low-occupancy regions coinciding with the host lattice positions. This coexistence of a mobile carrier sublattice with a structurally stable host lattice provides direct dynamical evidence of sublattice melting: the carrier

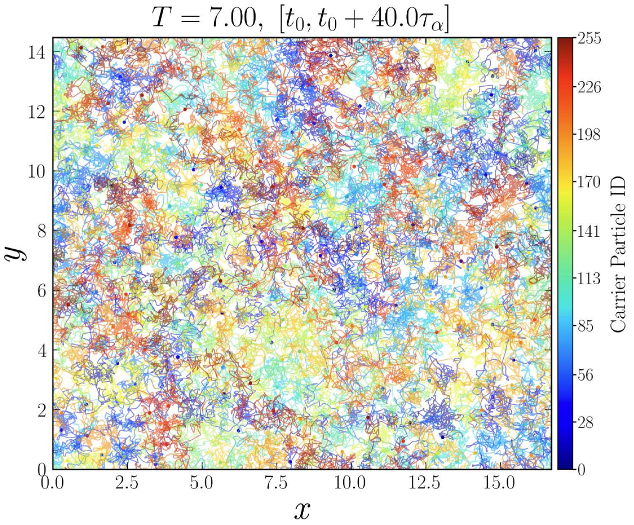

Fig. S14. Movie S1: In reference to Fig. 3*A,B* from the main text, carrier trajectories at $T = 7.00$ show homogeneous, liquid-like motion due to melting of both carrier and host sublattices. Movie link is here: https://doi.org/10.5281/zenodo.20482338.

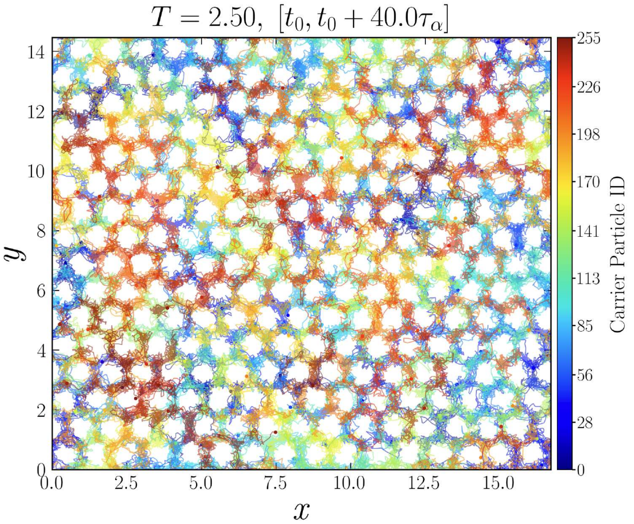

Fig. S15. Movie S2: In reference to Fig. 3*C,D* from the main text, at $T = 2.50$, the carrier sublattice is molten while the host lattice remains rigid, leading to bottleneck-guided but system-spanning transport. Movie link is here: https://doi.org/10.5281/zenodo.20482338.

sublattice is fully melted and percolates through the system, whereas the host lattice retains its positional order. The resulting heterogeneous flow pathways highlight the constrained nature of carrier transport imposed by the rigid host framework, even at elevated temperatures.

References

1. O Kamishima, Y Iwai, T Hattori, K Kawamura, J Kawamura, Vibrational analysis of ion dynamics in Ag β-alumina by Raman and molecular dynamics simulation. *J. Phys. Soc. Jpn.* **79**, 33–36 (2010).
2. F Takeiri, et al., Hydride-ion-conducting K_2NiF_4-type Ba–Li oxyhydride solid electrolyte. *Nat. Mater.* **21**, 325–330 (2022).
3. K Funke, Solid state ionics: from Michael Faraday to green energy—the European dimension. *Sci. Technol. Adv. Mater.* **14**, 043502 (2013).
4. S Torquato, Hyperuniform states of matter. *Phys. Reports* **745**, 1–95 (2018).
5. K Zahn, R Lenke, G Maret, Two-stage melting of paramagnetic colloidal crystals in two dimensions. *Phys. Rev. Lett.* **82**, 2721–2724 (1999).
6. X Li, et al., Hopping rate and migration entropy as the origin of superionic conduction within solid-state electrolytes. *J. Am. Chem. Soc.* **145**, 11701–11709 (2023).
7. T Krauskopf, C Pompe, MA Kraft, WG Zeier, Influence of lattice dynamics on Na^+ transport in the solid electrolyte $Na_3PS_{4-x}Se_x$. *Chem. Mater.* **29**, 8859–8869 (2017).
